# Psychological effects and associated factors among vaccinated and unvaccinated general population against COVID-19 infection in Bangladesh

**DOI:** 10.3389/fpsyt.2022.916160

**Published:** 2022-08-12

**Authors:** Md. Dhedharul Alam, Md. Joynal Abedin, Asraful Islam, Md. Mosfeq-Ul-Hasan, Obaydur Rahman, Yi Xu

**Affiliations:** ^1^Department of Psychiatry, The First Affiliated Hospital, Zhejiang University School of Medicine, Hangzhou, China; ^2^The Key Laboratory of Mental Disorder Management in Zhejiang Province, Hangzhou, China; ^3^Department of Population Sciences, University of Dhaka, Dhaka, Bangladesh; ^4^Department of Psychology, Jagannath University, Dhaka, Bangladesh; ^5^Examination Controller Section, Hajee Mohammad Danesh Science and Technology University, Dinajpur, Bangladesh; ^6^Department of Physics, Mawlana Bhashani Science and Technology University, Tangail, Bangladesh

**Keywords:** Bangladesh, COVID-19, general populations, immunization, psychological effects, refusal, uptake

## Abstract

**Background:**

The global effort to develop herd immunity in the general public against the COVID-19 pandemic is currently ongoing. However, to the best of our knowledge, there have been no studies on how the COVID-19 vaccine affects mental health in the context of the COVID-19 pandemic in Bangladesh. The present study investigated the psychological effects and associated factors among vaccinated and unvaccinated general populations against COVID-19 infection in Bangladesh.

**Methods:**

A nationwide online cross-sectional survey was conducted in Bangladesh from June 23 to December 25, 2021. The frequency of symptoms of psychological distress, depression, anxiety, stress, post-traumatic stress disorder (PTSD), insomnia, and fear was assessed using the Bangla versions of the GHQ-12, PHQ-2, GAD-2, PSS-4, PC-PTSD-5, ISI, and FCV-19S scales, respectively.

**Results:**

The study included 3,013 persons from all eight divisions of Bangladesh, with 1,272 (42.2%) being vaccinated and 1,741 (57.8%) being unvaccinated. Compared with unvaccinated populations, vaccinated populations had significantly lower prevalence rates of psychological distress (36.4 vs. 51.5%), depression (21.1 vs. 37.9%), anxiety (25.1 vs. 44.9%), stress (19.4 vs. 30.4%), PTSD (29.4 vs. 38.3%), insomnia (18.7 vs. 39.4%), and fear symptoms (16.1 vs. 27.5%). Among vaccinated populations, respondents who lived in nuclear families were significantly associated with higher risk of psychological distress (AOR, 1.38; 95% CI, 1.09–1.78), depression (AOR, 1.49; 95% CI, 1.11–1.98), anxiety (AOR, 1.77; 95% CI, 1.21–1.98), and fear (AOR, 1.43; 95% CI, 1.11–1.83) symptoms. Participants who lost family members, friends, or colleagues due to the COVID-19 pandemic had significantly higher risk of symptoms of psychological distress (AOR, 1.35; 95% CI, 1.02–1.79), anxiety (AOR, 1.41; 95% CI, 1.11–1.87), and PTSD (AOR, 1.76; 95% CI, 1.24–2.19). On the other hand, unvaccinated populations who lived in the Dhaka division were significantly associated with an increased risk of depression (AOR, 2.06; 95% CI, 1.40–2.52), anxiety (AOR, 1.86; 95% CI, 1.15–2.47), stress (AOR, 1.92; 95% CI, 1.12–2.88), and insomnia (AOR, 1.88; 95% CI, 1.20–2.94) symptoms. Except for PTSD and fear symptoms, unemployed participants had considerably higher rates of psychological distress, depression, anxiety, stress, and insomnia symptoms (e.g., psychological distress: AOR, 1.83; 95% CI, 1.10–2.62; depression: AOR, 1.74; 95% CI, 1.37–2.19).

**Conclusions:**

This study recommends immunizing unvaccinated populations as soon as possible to prevent infection and boost mental health. Vulnerable people needed special care, health-related education, and psychological assistance.

## Introduction

Since the commencement of the Coronavirus Disease 2019 (COVID-19) pandemic in 2019, more than 536 million people from 225 countries have been infected with the virus. Approximately 6.3 million people passed away (as of June 19, 2022) (1). To prevent the epidemic from spreading further, all governments have established mandatory measures including containment, quarantine, community control, and business and school closures (2–4). As a result of this large-scale contagious public health calamity and significant disruptions in daily life, people are under a lot of stress. They are experiencing a lot of mental health problems (5). In previous epidemiological studies, survivors of the severe acute respiratory syndrome (SARS), middle east respiratory syndrome (MERS), and Ebola virus disease (EVD) outbreaks reported depression, anxiety, negative psychological repercussions, panic attacks, psychomotor excitement, psychotic symptoms, loneliness, boredom, delirium, and even suicidal tendencies (6–9). According to a recent comprehensive review study, the prevalence of depression, anxiety, and post-traumatic stress disorder (PTSD) symptoms among SARS survivors were 19, 20, and 28%, respectively (10).

However, the estimates of psychological problems among the general public are higher than those for regular periods during the COVID-19 pandemic (11, 12). A recent systematic review of global (including 32 different countries and 398,771 participants) prevalence of mental health issues in the general population showed prevalence's of psychological distress, depression, anxiety, stress, PTSD, and sleep problems, at 50.0, 28.0, 26.9, 36.5, 24.1, and 27.6%, respectively (13). Another review study included 82 studies with a total of 96,100 participants showed that the overall prevalence of depression (23.9%), anxiety (23.4%), stress (14.2%), distress (16.0%), PTSD (24.9%), insomnia (26.5%), and poor mental health (26.5%) during the SARS and COVID-19 epidemics (14). Bangladesh, a densely populated and resource-limited country is confronted with widespread devastation and serious psychological issues during the COVID-19 outbreak (15). The first COVID-19 case was reported in Bangladesh on March 8, 2020, and as of June 19, 2022, the country had 1.95 million verified COVID-19 cases and 29,131 deaths (Supplementary Figures S1, S2) (16). Bangladesh is among the top 32 countries contributing to 0.56% of the COVID-19 cases in the world.

Like many countries, Bangladesh has employed various tactics to limit the spread of COVID-19, including lockdown, social distancing, self-isolation, and quarantine (17). The government announced a nationwide lockdown from March 26 to May 30, 2020, which was extended seven times (18, 19). In addition, until August 31, 2020, the government has imposed limits on public activities and movement across the country to prevent the spread of COVID-19 (20). As a result of the virus's ongoing spread and pandemic-related limits, the general public is progressively experiencing various types of psychological distress. According to previous studies, many people acquired psychological symptoms during the COVID-19 pandemic, including depression, anxiety, stress, panic attacks, sleep problems, and even suicidal ideation (21–23). During the early stages of the COVID-19 pandemic, a statewide survey of 1,427 persons in Bangladesh found that 57.9% of participants experienced depression, 33.7% had anxiety, and 59.7% had stress (24).

Vaccination has quickly emerged as an important strategy for prevention in the current COVID-19 pandemic. High vaccination coverage rates are necessary to protect the entire population indirectly. The global economy will reopen, and society will resume its regular routines, which is especially important given the present COVID-19 pandemic (25). High vaccination rates are also required to develop herd immunity, which minimizes COVID-19 transmission and infection risk in the general population and those most vulnerable to illness (26, 27). It has been estimated between 55 and 82% of populations would need to be vaccinated to reach herd immunity for COVID-19, depending on varying biological, environmental, socio-behavioral factors and infection rates within each country (28). Vaccine hesitancy, described as delayed acceptance, hesitation, or refusal of vaccination despite the availability of vaccination services, is a barrier to establishing herd immunity (27, 29).

The World Health Organization's strategy for achieving universal COVID-19 vaccination by mid-2022 lays out the path we must all take together to meet the goals of vaccinating 40% of the population of each country by the end of 2021 and 70% by the middle of 2022 (30). Bangladesh started distributing COVID-19 vaccinations on January 27, 2021, with bulk immunization beginning on February 7, 2021, and the second dosage starting on April 8, 2021. As of June 19, 2022, the number of first doses administered in Bangladesh exceeds 128,943,393 (75.7% of the total population), and the number of second doses administered exceeds 118,629,297 (69.7% of the total population) (Supplementary Figure S3) (31). With such a large number of volunteers being vaccinated, their psychological wellbeing should be examined as well. Even though COVID-19 vaccinations are safe for most persons over the age of 18, uncommon adverse effects still occur. After immunization, moderate side effects such as arm discomfort, slight fever, weariness, and headaches have been noted (32, 33). Furthermore, vaccine efficacy had not been well-validated in the general public before mass immunizations, and controversy about efficacy lingered even among those vaccinated (34).

A nationally representative Understanding America Study (UAS) of 8,003 adults in the United States discovered that people experienced lower distress levels after receiving the first dose of the COVID vaccination (35). Another study conducted in China discovered that stress levels dramatically lowered after vaccination (36). Moreover, a study conducted among 1,779 adults in Germany between January 1, 2021, to January 11, 2021, showed that COVID-19 vaccination could positively correlate with COVID-19-related anxiety and fears (37). Furthermore, a survey of 250 Jordanians who received their first dose of the COVID-19 vaccine by Al-Amer et al. (38) revealed that the vaccine is a source of distress for those who receive it for the first time, with higher levels of stress and anxiety after vaccination among those who experienced normal levels of anxiety before vaccination. Individuals with certain conditions (e.g., chronic disease such as psoriasis) as well as those intensively exposed to vaccine-related conspiracy beliefs may develop distress symptoms following vaccination. However, to our knowledge, there have been no studies on the psychological effects of COVID-19 vaccination among both vaccinated and unvaccinated general populations in Bangladesh yet. Therefore, we conducted a cross-sectional nationwide survey to assess the psychological effects and associated factors among the vaccinated and unvaccinated general population against SARS-CoV-2 infection in Bangladesh. The goal of this study opted to examine the prevalence of psychological distress, depression, anxiety, stress, PTSD, insomnia, and fear symptoms among the vaccinated and unvaccinated people against SARS-CoV-2 infection in Bangladesh and explored its contributing factors. This research will add to our understanding, describe, and address the general public's change in psychological effects after receiving the COVID-19 vaccine. It may also assist the government and policymakers in providing comprehensive and accurate information to those who are hesitant or resistant to vaccination and boost their confidence in the ongoing vaccination campaign.

## Materials and methods

### Study design

A cross-sectional design was utilized to perform this study. The study protocol was reviewed and approved by the Department of Psychology, Jagannath University, Dhaka, Bangladesh (JnU/DoP/206021), and the Ethics Committee of the First Affiliated Hospital, Zhejiang University School of Medicine, Hangzhou, China (IIT20220190B). The criteria for inclusion were as follows: (1) possessing the following requirements: being at least 18 years old, (2) residing in Bangladesh at the time of the COVID-19, (3) being willing to participate in this study by providing online informed consent, (4) completing the entire questionnaire, (5) not having a history of mental health issues, and (6) getting the second dose of the COVID-19 vaccines.

### Participants

The sample size was calculated using OpenEpi software. A prior investigation of the SARS-CoV-2 outbreak in Bangladesh discovered that about half of the people had psychological issues (24). This 50% proportion would provide maximum variance and sample size. At 95% confidence level, 80% power, and 2.5 design effect, we arrived at a sample size of 960. The estimated sample was 1,920, assuming an equal number of respondents (*n* = 960) in the vaccinated and unvaccinated subsamples. However, assuming a non-response rate of 10%, the final sample size decided in the current study was 2,112, with 1,056 respondents in each subsample.

### Procedure

A nationwide online study using Google Forms and the Bangla language was done from June 23 to December 25, 2021. The goal of this study was mentioned on the first page of the online form, and all participants' consent was ensured on that page. The five research assistants distributed the poll link *via* e-mail, Facebook, Viber, WhatsApp, Imo, and other social media sites. Participants were encouraged to complete the form and share the link with their networks to reach a larger audience. The five research assistants used convenient and snowball sampling to circulate the survey link throughout their professional and social networks. Participants were instructed that participating in the study was completely voluntary and that they should share the survey link with their colleagues or friends once they finished it. All participants were given assurances about the privacy and confidentiality of their information and the ability to have their data removed at any time. The current study received a total of 3,064 responses at the onset. After screening, 51 responses were eliminated due to incomplete information, the first dose vaccinated, under 18 years old, and being outside of Bangladesh. Finally, responses from 3,013 general populations were included in this study. A total of 3,013 Bangladeshi people completed the questionnaire, with 1,272 (42.2%) vaccinated and 1,741 (57.8%) unvaccinated.

### Measurements

#### Demographic, health, and COVID-19-related information

The participant's sex (male or female), age (18–29, 30–39, 40–49, 50–59 or ≥60 years), divisions (Dhaka, Chittagong, Barisal, Khulna, Rajshahi, Rangpur, Mymensingh, or Sylhet), residence (urban or rural), nature of family (nuclear or joint), educational level (college/below or university/higher) were self-reported demographic characteristics. Participants were asked about their status of marriage, whether or not they had children, occupation (student, unemployed, employed, businessman, housewife, or other), and socioeconomic status (lower, middle or upper class). In addition, participants were also asked to provide health, behavior, and COVID-19-related information (yes or no). Such as daily physical exercise, smoking habit, current alcohol drinking behavior, daily social media use, whether participants had been infected with COVID-19, whether anyone in their family members, friends, or colleagues had been infected with COVID-19, and whether anyone in their family members, friends, or colleagues had died from COVID-19.

#### General health questionnaire

The Bangla version of the twelve-item General Health Questionnaire (GHQ-12) (39, 40) evaluates psychological distress on a four-point Likert scale, with “1” defining never and “4” defining frequently. For a full score of 0 to 12, each item can be assigned a value of 0 (if option 1 or 2) or 1 (if options 3 and 4). An overall score ≥ 3 indicates a clinically significant level of poor mental health status. Its reliability in the current sample is very good (coefficient alpha = 0.80).

#### Patient health questionnaire

The Bangla version of the two-item Patient Health Questionnaire (PHQ-2) (41, 42) evaluates depression symptoms rated on a four-point Likert scale, with “1” defining never and “4” defining almost every day. The total score ranges from 0 to 6. An overall score ≥ 3 indicates a clinically significant level of depression. Its reliability in the present study is acceptable (coefficient alpha = 0.72).

#### Generalized anxiety disorder scale

The Bangla version of the two-item Generalized Anxiety Disorder scale (GAD-2) (43, 44) evaluates anxiety symptoms on a four-point Likert scale, with “1” defining never and “4” defining almost every day. The total score ranges from 0 to 6. An overall score ≥ 3 indicates a clinically significant level of anxiety. Its reliability in the current sample is very good (coefficient alpha = 0.84).

#### Perceived stress scale

The Bangla version of the four-item Perceived Stress Scale (PSS-4) (45, 46) evaluates stress symptoms on a four-point Likert scale, with “1” defining never and “4” defining always. The total score ranges from 0 to 16. An overall score ≥ 9 indicates a clinically significant level of stress. Its reliability in the present study is acceptable (coefficient alpha = 0.71).

#### Primary care PTSD screen for DSM-5

The Bangla version of the five-item Primary Care PTSD Screen for DSM-5 (PC-PTSD-5) (47, 48) evaluates post-traumatic stress disorder symptoms over the past month by asking five binary questions about re-experiencing, avoidance, physiological reactions, emotional numbness, and trauma-distorted guilt and blame thoughts. This scale was previously used in a Bangladeshi study. The total score ranges from 1 to 5. An overall score ≥ 3 indicates a clinically significant level of post-traumatic stress disorder. Its reliability in the current sample is acceptable (coefficient alpha = 0.75).

#### Insomnia severity index

The Bangla version of the seven-item Insomnia Severity Index (ISI) (49, 50) evaluates the severity of insomnia on a five-point Likert scale, with “0” defining no problem and “4” defining a major problem. The total score ranges from 0 to 28. An overall score ≥ 8 indicates a clinically significant level of insomnia. Its reliability in the present study is acceptable (coefficient alpha = 0.74).

#### Fear of COVID-19 scale

The Bangla version of the seven-item Fear of COVID-19 Scale (FCV-19S) (51, 52) evaluates the level of fear associated with COVID-19 on a five-point Likert scale, with “1” defining strongly disagree and “5” defining strongly agree. The total score ranges from 7 to 35. An overall score ≥ 18 indicates a clinically significant level of COVID-19-related fear. Its reliability in the current sample is very good (coefficient alpha = 0.89).

#### Oslo social support scale

The Bangla version of the three-item Oslo Social Support Scale (OSSS-3) evaluates respondents' social support (48, 53). The sum score ranges from 3 to 14, with high values representing strong levels and low values representing poor levels of social support. Social support has been leveled as poor, moderate, or strong based on a score of 3–8, 9–11, or 12–14. The reliability of the OSSS-3 in this study is acceptable (coefficient alpha = 0.79).

### Statistical analysis

The statistical analyses were run by SPSS version 20.0, and figures were prepared in GraphPad Prism version 9. Categorical data was represented using numbers and percentages. To compare categorical variable variations between groups, Chi-square tests were used. The Kolmogorov–Smirnov test, the Shapiro–Wilk test, and normal Q-Q plots were used to determine the data's normality. The median of the interquartile range (IQR) of data from non-normal distributions was shown. When comparing non-normally distributed data between two groups, the Mann–Whitney *U*-test was used, and when comparing data between more than two groups, the Kruskal–Wallis-test was used. Spearman correlations were used to compare the psychological effects of vaccinated and unvaccinated populations. In addition, binary logistic regression analysis was used to examine potential predictors of psychological effects in both groups. The model fitness test was checked using the Hosmer and Lemeshow goodness of fit test. All of the variables were added in the univariate analysis. Then the multivariate analysis only included the significant variables in the univariate analysis after controlling for confounders (e.g., sex, age, divisions, residence, etc.). For a single predictor, univariate analysis expressed as crude odds ratio (COR) was used, while multivariate analysis expressed as adjusted odds ratio (AOR) was used for multiple predictors, and psychological effects were considered dependent variables. All analyses were conducted at a 95% confidence level, with *p*-values equal to or <0.05 considered significant.

## Results

### Demographic, health, and COVID-19-related characteristics

Finally, 3,013 general populations were enrolled in our study, with 1,272 (42.2%) being vaccinated and 1,741 (57.8%) being unvaccinated. The demographic, health, and COVID-19-related characteristics of the study participants are shown in Table 1. Vaccinated populations were significantly more likely to be Dhaka division (56.8 vs. 28.9%, *p* = 0.00), be married (75.5 vs. 65.8%, *p* = 0.00), having children (48.3 vs. 38.1%, *p* = 0.00), smoke (33.5 vs. 24.0%, *p* = 0.00), have chronic diseases (31.1 vs. 7.9%, *p* = 0.00), daily social media used (43.3 vs. 29.4%, *p* = 0.00), be infected with COVID-19 (39.5 vs. 26.3%, *p* = 0.00), have family members, friends, or colleagues infected with COVID-19 (45.8 vs. 32.1%, *p* = 0.00), have family members, friends, or colleagues died from COVID-19 (32.2 vs. 24.6%, *p* = 0.00), and moderate social support (60.9 vs. 45.1%, *p* = 0.00) than unvaccinated populations. On the other hand, unvaccinated populations were significantly more likely to be male (66.2 vs. 56.4%, *p* = 0.00), 30–39 years old (60.0 vs. 58.8%, *p* = 0.00), and have a joint family (64.9 vs. 60.5%, *p* = 0.02) than vaccinated populations. However, there were no significant differences between the vaccinated and unvaccinated populations in terms of residence (*p* = 0.06), education level (*p* = 0.06), occupation (*p* = 0.17), socioeconomic status (*p* = 0.61), physical exercise (*p* = 0.52), and current alcohol drinking behavior (*p* = 0.18).

**Table 1:** Demographic, health, and COVID-19-related characteristics in vaccinated and unvaccinated populations against COVID-19 infection.

**Characteristics**	**Total** **(*n* = 3,013)**	**Vaccinated population** **(*n* = 1,272)**	**Unvaccinated** **population (*n* = 1,741)**	**χ^2^**	**df**	***p-*value**
	**No. (%)**	**No. (%)**	**No. (%)**			
**Sex**						
Male	1,870 (62.1)	718 (56.4)	1,152 (66.2)	29.50	1	0.00
Female	1,143 (37.9)	554 (43.6)	589 (33.8)			
**Age, y**						
18–29	448 (14.9)	166 (13.1)	282 (16.2)	14.26	4	0.00
30–39	1,792 (59.5)	748 (58.8)	1,044 (60.0)			
40–49	647 (21.5)	293 (23.0)	354 (20.3)			
50–59	96 (3.2)	53 (4.2)	43 (2.5)			
≥60	30 (1.0)	12 (0.9)	18 (1.0)			
**Divisions of Bangladesh**						
Dhaka	1,226 (40.7)	722 (56.8)	504 (28.9)	254.93	7	0.00
Chittagong	415 (13.8)	152 (11.9)	263 (15.1)			
Barisal	294 (9.8)	80 (6.3)	214 (12.3)			
Khulna	365 (12.1)	129 (10.1)	236 (13.6)			
Rajshahi	196 (6.5)	50 (3.9)	146 (8.4)			
Rangpur	221 (7.3)	56 (4.4)	165 (9.5)			
Mymensingh	115 (3.8)	25 (2.0)	90 (5.2)			
Sylhet	181 (6.0)	58 (4.6)	123 (7.1)			
**Residence**						
Urban	1,805 (59.9)	737 (57.9)	1,068 (61.3)	3.54	1	0.06
Rural	1,208 (40.1)	535 (42.1)	673 (38.7)			
**Family type**						
Nuclear	1,114 (37.0)	503 (39.5)	611 (35.1)	6.24	1	0.02
Joint	1,899 (63.0)	769 (60.5)	1,130 (64.9)			
**Education level**						
College/below	1,069 (35.5)	427 (33.6)	642 (36.9)	3.51	1	0.06
University/higher	1,944 (64.5)	845 (66.4)	1,099 (63.1)			
**Marital status**						
Single	716 (23.8)	258 (20.3)	458 (26.3)	36.76	2	0.00
Married	2,105 (69.9)	960 (75.5)	1,145 (65.8)			
Divorced/separated/widowed	192 (6.4)	54 (4.2)	138 (7.9)			
**Having children**						
Yes	1,279 (42.4)	615 (48.3)	664 (38.1)	31.36	1	0.00
No	1,734 (57.6)	657 (51.7)	1,077 (61.9)			
**Occupation**						
Student	227 (7.5)	103 (8.1)	124 (7.1)	7.76	5	0.17
Unemployed	156 (5.2)	72 (5.7)	84 (4.8)			
Employed	1,587 (52.7)	652 (51.3)	935 (53.7)			
Businessman	347 (11.5)	143 (11.2)	204 (11.7)			
Housewife	102 (3.4)	54 (4.2)	48 (2.8)			
Other	594 (19.7)	248 (19.5)	346 (19.9)			
**Socioeconomic status**						
Lower	208 (6.9)	87 (6.8)	121 (7.0)			
Middle	801 (26.6)	350 (27.5)	451 (25.9)	0.97	2	0.61
Upper	2,004 (66.5)	835 (65.6)	1,169 (67.1)			
**Physical exercise**						
Yes	825 (27.4)	356 (28.0)	469 (26.9)	0.40	1	0.52
No	2,188 (72.6)	916 (72.0)	1,272 (73.1)			
**Smoking habit**						
Yes	843 (28.0)	426 (33.5)	417 (24.0)	33.18	1	0.00
No	2,170 (72.0)	846 (66.5)	1,324 (76.0)			
**Alcohol use**						
Yes	163 (5.4)	77 (6.1)	86 (4.9)	1.78	1	0.18
No	2,850 (94.6)	1,195 (93.9)	1,655 (95.1)			
**Chronic diseases**						
Yes	533 (17.7)	395 (31.1)	138 (7.9)	269.98	1	0.00
No	2,480 (82.3)	877 (68.9)	1,603 (92.1)			
**Social media use**						
Yes	1,062 (35.2)	551 (43.3)	511 (29.4)	62.81	1	0.00
No	1,951 (64.8)	721 (56.7)	1,230 (70.6)			
**Have you been infected with COVID-19?**						
Yes	961 (31.9)	503 (39.5)	458 (26.3)	59.29	1	0.00
No	2,052 (68.1)	769 (60.5)	1,283 (73.7)			
**Have any of your family members, friends, or colleagues been infected with COVID-19?**						
Yes	1,141 (37.9)	582 (45.8)	559 (32.1)	58.17	1	0.00
No	1,872 (62.1)	690 (54.2)	1,182 (67.9)			
**Have any of your family members, friends, or colleagues died of COVID-19?**						
Yes	839 (27.8)	410 (32.2)	429 (24.6)	21.08	1	0.00
No	2,174 (72.2)	862 (67.8)	1,312 (75.4)			
**Social support**						
Poor	911 (30.2)	330 (25.9)	581 (33.4)	77.92	2	0.00
Moderate	1,560 (51.8)	775 (60.9)	785 (45.1)			
Strong	542 (18.0)	167 (13.1)	375 (21.5)			

### Scores of psychological effects

Table 2 shows the median of the IQR of psychological effects scores in vaccinated and unvaccinated populations against COVID-19 infection. When compared to unvaccinated populations, vaccinated populations had significantly lower median of the IQR of scores for depression (1.0 [1.0–2.0] vs. 3.0 [1.0–4.0]; *p* = 0.00), anxiety (2.0 [1.0–3.0] vs. 3.0 [1.0–4.0]; *p* = 0.00), stress (1.0 [7.0–8.0] vs. 6.0 [4.0–10.0]; *p* = 0.00), insomnia (3.0 [4.0–7.0] vs. 7.0 [4.0–11.0]; *p* = 0.00), and fear (7.0 [9.0–16.0] vs. 10.0 [9.0–19.0]; *p* = 0.01) symptoms, but significantly same median of the IQR of scores for psychological distress symptoms. However, the IQR of scores for PTSD symptoms did not differ significantly between vaccinated and unvaccinated populations (*p* = 0.23).

**Table 2:** The median of the interquartile range (IQR) of psychological effects scores in vaccinated and unvaccinated populations against COVID-19 infection.

**Psychological effects**	**Vaccinated population**	**Unvaccinated population**	**z score**	***p-*value**
	**Median (IQR)**	**Median (IQR)**		
Psychological distress symptoms	5.0 (1.0–6.0)	5.0 (1.0–6.0)	−5.64	0.00
Depression symptoms	1.0 (1.0–2.0)	3.0 (1.0–4.0)	−12.4	0.00
Anxiety symptoms	2.0 (1.0–3.0)	3.0 (1.0–4.0)	−7.61	0.00
Stress symptoms	1.0 (7.0–8.0)	6.0 (4.0–10.0)	−10.5	0.00
PTSD symptoms	3.0 (1.0–4.0)	3.0 (1.0–4.0)	−1.19	0.23
Insomnia symptoms	3.0 (4.0–7.0)	7.0 (4.0–11.0)	−12.6	0.00
Fear symptoms	7.0 (9.0–16.0)	10.0 (9.0–19.0)	−2.51	0.01

IQR, Interquartile range; PTSD, Post-traumatic stress disorder.

### Prevalence of psychological effects

**Figure 1:**
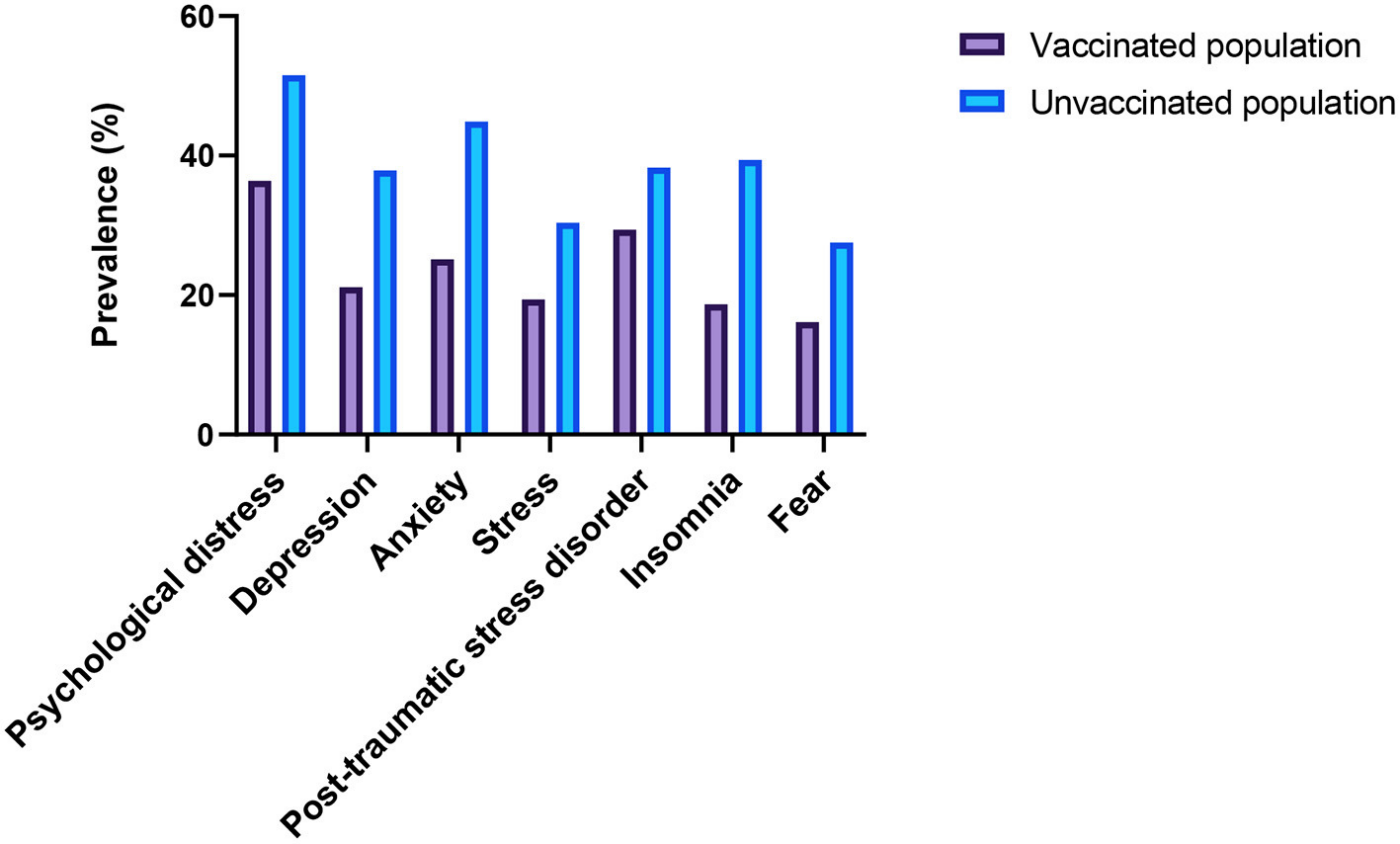
Prevalence of psychological effects among the vaccinated and unvaccinated populations against COVID-19 infection.

The prevalence of psychological effects among vaccinated and unvaccinated populations against COVID-19 infection are shown in Figure 1 and Table 3. The prevalence rates of symptoms of psychological distress, depression, anxiety, stress, PTSD, insomnia, and fear symptoms among vaccinated populations were 36.4, 21.1, 25.1, 19.4, 29.4, 18.7, and 16.1%, respectively. On the other hand, the prevalence rates of symptoms of psychological distress, depression, anxiety, stress, PTSD, insomnia, and fear symptoms among unvaccinated populations were 51.5, 37.9, 44.9, 30.4, 38.3, 39.4, and 27.5%, respectively. However, these findings showed that vaccinated populations had significantly lower prevalence rates of psychological distress (36.4 vs. 51.5%, *p* = 0.00), depression (21.1 vs. 37.9%, *p* = 0.00), anxiety (25.1 vs. 44.9%, *p* = 0.00), stress (19.4 vs. 30.4%, *p* = 0.00), PTSD (29.4 vs. 38.3%, *p* = 0.00), insomnia (18.7 vs. 39.4%, *p* = 0.00), and fear symptoms (16.1 vs. 27.5%, *p* = 0.00) than unvaccinated populations.

**Table 3:** The prevalence of psychological effects among vaccinated and unvaccinated populations against COVID-19 infection.

**Psychological effects**	**Total (*n* = 3,013)**	**Vaccinated population** **(*n* = 1,272)**	**Unvaccinated** **population (*n* = 1,741)**	***p-*value**
	**No. (%)**	**No. (%)**	**No. (%)**	
**Psychological distress symptoms**				
Yes	1,359 (45.1)	463 (36.4)	896 (51.5)	0.00
No	1,654 (54.9)	809 (63.6)	845 (48.5)	
**Depression symptoms**				
Yes	928 (30.8)	268 (21.1)	660 (37.9)	0.00
No	2,085 (69.2)	1,004 (78.9)	1,081 (62.1)	
**Anxiety symptoms**				
Yes	1,101 (36.5)	319 (25.1)	782 (44.9)	0.00
No	1,912 (63.5)	953 (74.9)	959 (55.1)	
**Stress symptoms**				
Yes	776 (25.8)	247 (19.4)	529 (30.4)	0.00
No	2,237 (74.2)	1,025 (80.6)	1,212 (69.6)	
**Post-traumatic stress disorder symptoms**				
Yes	1,040 (34.5)	374 (29.4)	666 (38.3)	0.00
No	1,973 (65.5)	898 (70.6)	1,075 (61.7)	
**Insomnia symptoms**				
Yes	924 (30.7)	238 (18.7)	686 (39.4)	0.00
No	2,089 (69.3)	1,034 (81.3)	1,055 (60.6)	
**Fear symptoms**				
Yes	684 (22.7)	205 (16.1)	479 (27.5)	0.00
No	2,329 (77.3)	1,067 (83.9)	1,262 (72.5)	

### Correlations of psychological effects

Spearman's correlations of psychological effects among vaccinated and unvaccinated populations are shown in Table 4. In the vaccinated populations, there was a positive correlation between psychological distress scores and depression (r_s_ = 0.118, *p* < 0.01) scores. Moreover, depression scores were positively linked to anxiety (r_s_ = 0.207, *p* < 0.01), and PTSD (r_s_ = 0.134, *p* < 0.01) scores. Furthermore, there was a positive relationship between anxiety and PTSD (r_s_ = 0.117, *p* < 0.01) scores. In the unvaccinated populations, there was a positive correlation between psychological distress scores and fear (r_s_ = 0.139, *p* < 0.01) scores. Moreover, depression scores were positively linked to anxiety (r_s_ = 0.306, *p* < 0.01) and insomnia (r_s_ = 0.762, *p* < 0.01) scores. Furthermore, there was a positive relationship between anxiety scores and stress (r_s_ = 0.825, *p* < 0.01), PTSD (r_s_ = 0.212, *p* < 0.01), and insomnia (r_s_ = 0.644, *p* < 0.01) scores. In addition, stress scores were positively linked to PTSD (r_s_ = 0.832, *p* < 0.01) and insomnia (r_s_ = 0.773, *p* < 0.01) scores.

**Table 4:** Spearman's correlations of psychological effects among vaccinated and unvaccinated populations against COVID-19 infection.

**Populations**	**Psychological effects**	**1**	**2**	**3**	**4**	**5**	**6**	**7**
Vaccinated population	1	1.00						
	2	0.118^**^	1.00					
	3	0.003	0.207^**^	1.00				
	4	0.053	0.011	0.005	1.00			
	5	0.002	0.134^**^	0.117^**^	0.014	1.00		
	6	−0.011	0.044	0.004	0.018	0.030	1.00	
	7	−0.019	−0.014	0.006	0.004	0.005	0.018	1.00
Unvaccinated population	1	1.00						
	2	0.038	1.00					
	3	0.014	0.306^**^	1.00				
	4	0.016	0.031	0.825^**^	1.00			
	5	0.006	0.025	0.212^**^	0.832^**^	1.00		
	6	0.003	0.762^**^	0.644^**^	0.773^**^	0.042	1.00	
	7	0.139^**^	0.003	0.002	0.028	0.024	0.014	1.00

** p < 0.01. 1. Psychological distress, 2. Depression, 3. Anxiety, 4. Stress, 5. Post-traumatic stress disorder, 6. Insomnia, 7. Fear.

### Factors associated with psychological effects

The univariate logistic regression analysis results are presented in Supplementary Table S1. The multivariate logistic regression analysis (Supplementary Table S2) showed that among the COVID-19 vaccine recipients, males were significantly higher risk of symptoms of depression (AOR, 1.80; 95% CI, 1.14–2.14), anxiety (AOR, 1.79; 95% CI, 1.13–2.04), and PTSD (AOR, 1.49; 95% CI, 1.18–1.93) compared to females. Vaccinated respondents who lived in nuclear families were significantly higher risk of symptoms of psychological distress (AOR, 1.38; 95% CI, 1.09–1.77), depression (AOR, 1.49; 95% CI, 1.11–1.98), anxiety (AOR, 1.69; 95% CI, 1.21–1.98), and fear (AOR, 1.43; 95% CI, 1.11–1.82) than those who lived in joint families. Except for stress and fear symptoms, married vaccine recipients people were significantly greater risk of all psychological symptoms than those who were divorced, separated, or widowed (e.g., psychological distress: AOR, 1.44; 95% CI, 1.08–1.90; depression: AOR, 2.37; 95% CI, 1.72–3.24). Vaccinated people who used daily social media had significantly more likely to suffer from symptoms of psychological distress (AOR, 1.75; 95% CI, 1.18–2.01), depression (AOR, 1.48; 1.21–1.83), anxiety (AOR, 1.57; 95% CI, 1.16–1.87), and insomnia (AOR, 1.68; 95% CI, 1.02–1.97). Vaccinated respondents who had COVID-19 infected family members, friends, or colleagues were considerably more likely to experience symptoms of psychological distress (AOR, 2.01; 95% CI, 1.33–2.86) and PTSD (AOR, 1.59; 95% CI, 1.10–1.86), but were less likely to experience symptoms of depression (AOR, 0.76; 95% CI, 0.47–0.94) and anxiety (AOR, 0.54; 95% CI, 0.21–0.81). Vaccinated people who lost family members, friends, or colleagues due to the COVID-19 pandemic had significantly higher risk of symptoms of psychological distress (AOR, 1.35; 95% CI, 1.02–1.79), anxiety (AOR, 1.41; 95% CI, 1.11–1.87), and PTSD (AOR, 1.76; 95% CI, 1.24–2.19) than those who did not.

On the other hand, unvaccinated populations who lived in the Dhaka division were significantly associated with an increased risk of depression (AOR, 2.06; 95% CI, 1.40–2.52), anxiety (AOR, 1.86; 95% CI, 1.15–2.47), stress (AOR, 1.92; 95% CI, 1.12–2.88), and insomnia (AOR, 1.88; 95% CI, 1.20–2.94) symptoms than those living in other divisions. Unvaccinated people who had children had significantly higher risk of depression (AOR, 1.66; 95% CI, 1.21–2.18), anxiety (AOR, 1.49; 95% CI, 1.12–1.87), stress (AOR, 1.43; 95% CI, 1.07–1.83), and PTSD (AOR, 1.32; 95% CI, 1.05–1.71) symptoms than those who did not. Except for PTSD and fear symptoms, unvaccinated participants who were unemployed had considerably higher rates of psychological distress (AOR, 1.83; 95% CI, 1.10–2.62), depression (AOR, 1.74; 95% CI, 1.37–2.19), anxiety (AOR, 1.70; 95% CI, 1.12–2.11), stress (AOR, 1.46; 95% CI, 1.08–1.92), and insomnia (AOR, 1.87; 95% CI, 1.20–2.46) symptoms. Unvaccinated people who drank alcohol had significantly greater risk of symptoms of psychological distress (AOR, 2.01; 95% CI, 1.23–2.78), depression (AOR, 1.96; 95% CI, 1.17–2.41), anxiety (AOR, 2.28; 95% CI, 1.27–3.03), and stress (AOR, 1.83; 95% CI, 1.04–2.66) than those who did not. When compared to unvaccinated people who did not have chronic diseases, those with chronic diseases were significantly more likely to experience symptoms of depression (AOR, 1.75; 95% CI, 1.16–2.01), anxiety (AOR, 1.51; 95% CI, 1.07–1.82), and PTSD (AOR, 1.98; 95% CI, 1.12–2.41). Unvaccinated people who were infected with COVID-19 had considerably higher risk of psychological distress (AOR, 1.66; 95% CI, 1.23–2.02), depression (AOR, 1.88; 95% CI, 1.07–2.01), and anxiety (AOR, 1.61; 95% CI, 1.22–1.98) symptoms, but lower risk of fear (AOR, 0.69; 95% CI, 0.41–0.91) symptoms than those who did not. When compared to unvaccinated respondents who had strong social support, those who had poor social support had significantly greater experience of depression (AOR, 1.88; 95% CI, 1.35–2.41), anxiety (AOR, 1.48; 95% CI, 1.01–1.81), and PTSD (AOR, 1.71; 95% CI, 1.20–2.09) symptoms.

## Discussion

Bangladesh has been impacted heavily by the COVID-19 pandemic. Every sector has made it an important priority to reduce the impact of COVID-19. This is the first nationwide study that has evaluated the factors associated with psychological effects among vaccinated and unvaccinated populations against COVID-19 infection in Bangladesh. A total of 3,013 general populations were enrolled in our study, with 1,272 (42.2%) being vaccinated and 1,741 (57.8%) being unvaccinated. Our study showed lower prevalence of the symptoms of psychological distress, depression, anxiety, stress, PTSD, insomnia, and fear among vaccinated participants compared with those who were unvaccinated. Psychological distress and PTSD did not vary between groups all other symptoms were considerably lower among vaccinated participants.

This study showed that vaccinated populations had lower prevalence of psychological effects than unvaccinated populations against the COVID-19 outbreak in Bangladesh. Our results are consistent with a study conducted in China among 34,041 general public, which found that the psychological stress level decreased after vaccination (36). Moreover, a nationally representative cohort study of 5,792 adults conducted in the United States found that receiving a COVID-19 vaccination was linked to reduced psychological distress (54), which is also consistent with our findings. Furthermore, a study conducted in Turkey among 304 individuals by Bilge et al. (55) found that the vaccinated individuals had lower scores for depression and anxiety symptoms than unvaccinated individuals, indicating that vaccination may have a positive effect on improving mental health. This finding is also in line with our results. Understanding and addressing the general public's change in psychological effects after receiving the COVID-19 vaccine may assist the government and policymakers in providing comprehensive and accurate information to those who are hesitant or resistant to vaccination, as well as boosting their confidence in the ongoing vaccination campaign. The current study showed many demographic, health, and COVID-19-related factors linked to vaccinated and unvaccinated populations.

Our results showed that male vaccinated populations were significantly higher risk of symptoms of depression, anxiety, and PTSD compared to females, which is consistent with the findings in other studies (56). This finding is consistent with prior research, which found that male participants displayed a remarkably higher risk for depression symptoms (57). Another study found that male participants showed higher PTSD symptoms than females during the COVID-19 pandemic, which is also consistent with our results (58). But most of the previous Bangladeshi studies found that females were at a higher risk of depression, anxiety, and PTSD symptoms than males during the COVID-19 pandemic period (22, 24). Male participants' higher susceptibility to mental health symptoms during the pandemic may be due to their higher infective rate and more frequent risky behaviors during pandemics (59, 60). However, a study in Bangladesh reported that male participants were more likely to the willingness to pay for the COVID-19 vaccine than females, which is in line with our results (61). Males may have known more about COVID-19 vaccines in Bangladesh than females (62). Therefore, they are more likely to accept the COVID-19 vaccine.

The current study demonstrated that vaccinated people who lived in nuclear families were significantly higher risk of psychological distress, depression, anxiety, and fear symptoms. This finding is consistent with previous Bangladeshi research, which found that participants living in nuclear families reported a higher level of depression and anxiety symptoms during the COVID-19 pandemic (63). A nationwide cross-sectional study among 1,427 Bangladeshi adults found that individuals who lived in nuclear families (≤4 members) were more likely to suffer from psychological problems during the COVID-19 pandemic (24). Since the lockdown was implemented, it's probable that people have been in close contact with their families and have been forced to stay at home for extended periods. As a result, persons who lived in larger families were more likely to have meaningful dialogues and interactions with their family members than those who lived in nuclear households. Therefore, people who lived in nuclear families were more likely to suffer from mental illness. However, recent research in Bangladesh found that residing in a nuclear family was associated with more excellent knowledge about COVID-19 vaccines, which is consistent with our findings (64). Another study indicated that people who lived alone or with a large family (five people) were less likely to say they would be vaccinated for COVID-19 than people who lived in families of two to four people (65). People who grew up in nuclear households were probably more concerned about their health, so they were more accepting of vaccines.

Our findings found that except for stress and fear symptoms, vaccinated populations who were married were significantly associated with a greater risk of all psychological symptoms. A recent national cross-sectional study involving 1,311 community-dwelling individuals in Bangladesh, which found that participants who were married were more likely to be suffering from anxiety symptoms during the COVID-19 pandemic (23). Another study in the same country discovered that marriage increases the risk of mental health problems (66, 67). Both findings are consistent with our findings. It could be why married people have more family responsibilities than unmarried people. However, a cross-sectional online survey among 850 Bangladeshi adults discovered that married individuals were more aware of the vaccine than unmarried individuals, which is similar to our findings (68). Similarly, married people were more likely to declare their intention to obtain COVID-19 immunizations (69). The possible reason behind this might be that married people are worried about their partner. For both, immunization can minimize the chance of illness. Furthermore, knowledge distribution can be enough when they discuss with their partner. Perceived illness risks and attitudes alter depending on relationship status, influencing the decision to use a vaccine.

The present study discovered that vaccinated populations who used daily social media had significantly more likely to suffer from symptoms of psychological distress, depression, anxiety, and insomnia. A Bangladeshi study reported that respondents who used higher social media were associated with depression and anxiety symptoms, which is in line with our findings (70). Similar studies conducted in the same country also reported that those who follow COVID-19-related news on social or other media daily were more likely to have mental health problems (15, 21). Propaganda, falsehoods, conspiracy theories, and other aspects of the pandemic have risen, while social media has emerged as one of the most critical sources of COVID-19-related information. As a result, regularly utilizing social media was a substantial risk factor for psychological problems (71). In August 2020, a survey of 517 Nigerian social media users indicated that 74.5 percent intend to take the COVID-19 vaccination when it is ready (72). Similarly, Piltch-Loeb et al. (73) discovered that those who obtained vaccine information through traditional media rather than social media or both traditional and social media were more willing to accept it. Both studies corroborate our findings. This could be because social media platforms can help educate vaccination doubts, while traditional media outlets should continue to offer data-driven and informed vaccine information to their audiences.

Our findings revealed that vaccinated populations with COVID-19 infected family members, friends, or colleagues had significantly higher risk of symptoms of psychological distress and PTSD but lower risk of depression and anxiety. A study done on Bangladeshi residents demonstrated that participants who reported having family or acquaintances infected with COVID-19 were a protective factor against anxiety symptoms, confirming our findings (21). Furthermore, a cross-sectional study conducted on 3,480 people in Spain reported that those with a close relative infected were associated with more significant symptomatology in PTSD symptoms (74). Family members, acquaintances, or colleagues of COVID-19 patients may likely be concerned about becoming infected, quarantined, and feeling ostracized, all of which may worsen psychological problems. However, a large sample study in Bangladesh showed that those with family members diagnosed with COVID-19 were more likely to accept the COVID-19 vaccine, which is in line with our findings (75). Their decision was probably affected by social responsibility and positive experiences with vaccination and immunization programs.

The current study discovered that people who were vaccinated against COVID-19 and lost family members, friends, or coworkers were significantly higher risk of psychological distress, anxiety, and PTSD symptoms. In a study conducted in Bangladesh by Zubayer et al. (21), having relatives or acquaintances who died from COVID-19 was found to be a stronger predictor of anxiety symptoms, which is in line with our findings. Similarly, another study involving 10,754 people from 31 Iranian districts revealed that losing a loved one to COVID-19 causes psychological issues (76). This conclusion is also consistent with the framework proposed by Ghaleb et al. (66), which found that having a friend who died of COVID-19 was related to greater psychological distress levels. It could be due to people's concern for the wellbeing of family members, friends, or coworkers and their safety. However, a cross-sectional study of 883 people in Pakistan reported that those who had lost family members, friends, or coworkers significantly impacted their willingness to accept the COVID-19 vaccine (77). A probable explanation is that people whose relatives have died from COVID-19 may have learned more about the virus and its consequences on human health. As a result, they may desire to protect themselves by getting COVID-19 vaccines.

The present study found that unvaccinated populations who lived in the Dhaka division were significantly associated with an increased risk of depression, anxiety, stress, and insomnia symptoms. A study conducted among 10,609 individuals in the COVID-19 outbreak in Bangladesh showed that respondents who lived in Dhaka found higher experienced symptoms of depression, anxiety, and stress (78). According to two similar studies conducted in the same country, respondents who lived in the Dhaka division were significantly more likely to be depression, anxiety, and insomnia symptoms (22, 79). The initial findings supported our findings. This could be a contributing factor because the Dhaka division has the most significant population and handles most COVID-19 cases in Bangladesh (16). Therefore, during this or future pandemics, residents from the Dhaka division should receive special attention and care from the relevant authorities. In addition, Dhaka, the capital city of Bangladesh, is home to an estimated four million slum inhabitants (80). They are a socioeconomically deprived group with little understanding of COVID-19 and weak prophylactic actions against virus infection (17). This may contribute to their aversion to immunization. A comparable survey in the Mumbai slum in India reported a 20% unacceptance of COVID-19 vaccines among slum inhabitants (81).

Our findings showed that unvaccinated populations who had children had a significantly greater risk of depression, anxiety, stress, and PTSD symptoms. A recent study in Bangladesh found that respondents who had children during the COVID-19 pandemic had more depression and stress symptoms, which is confirmed by our findings (82). Similarly, it is also consistent with earlier studies conducted among the 1,041 general population of the Republic of Ireland, Karatzias et al. (58) revealed that people with children had a higher risk of PTSD symptoms. Similar findings are reported in a previous study (66). This link could be explained by concern about spreading the virus to family members. However, this outcome is consistent with other investigations, which found that vaccine hesitancy was higher among those who had children at home (83). It's possible that participants were worried about vaccine side effects on themselves or their children.

Unsurprisingly, the current study found that unvaccinated populations who were unemployed had higher rates of psychological distress, depression, anxiety, stress, and insomnia symptoms. This conclusion is backed by research of 974 healthy persons done in Bangladesh, which found that unemployed people had higher rates of depression, anxiety, and stress symptoms during the COVID-19 pandemic (84). This finding is also supported by a nationwide study conducted in the same country, which reported that unemployed respondents experienced high-stress levels during the COVID-19 pandemic (24). Similarly, an online cross-sectional survey among 672 Bangladeshi people during the COVID-19 pandemic showed that unemployed respondents were more likely to be had poor mental health (85). Unemployment is likely linked to low self-esteem, social isolation, and low income, which can contribute to psychological problems. However, a nationwide investigation in Bangladesh involving 1,134 adults aged 18 and over found that unemployment was associated with a higher risk of COVID-19 vaccine discomfort, which is consistent with our findings (86). On the other hand, other research found that unemployed persons were more inclined to receive the COVID-19 vaccination since, in some locations, unemployed people may seek to return to employment, which can only happen after immunization (83, 87).

The current study revealed that unvaccinated populations who drank alcohol had significantly higher risk of psychological distress, depression, anxiety, and stress symptoms. This conclusion is supported by several Bangladeshi COVID-19-related studies, which found that those who drank alcohol had a substantially increased risk of developing psychological problems (22, 88). Research shows that people who drink alcohol are more likely to develop mental health problems. People with severe mental illness are also more prone to have alcohol issues. This could be because they ‘self-medicate' or drink to cope with unpleasant feelings or symptoms (89). However, a nationwide study of 23,142 people in Japan found that respondents who consumed alcohol were significantly associated with COVID-19 vaccine hesitancy, which is similar to our results (90). Furthermore, a recent study demonstrated that those who drank alcohol daily had a lower level of vaccine literacy (91). It might be possible because there are misleading rumors and misconceptions about immunizations. To boost vaccine acceptance among the general population, our findings suggest that false rumors and misconceptions concerning COVID-19 immunizations should be eliminated, and people should be educated on the actual scientific facts.

The present study demonstrated that unvaccinated populations with chronic diseases were significantly higher risk of symptoms of depression, anxiety, and PTSD. Our findings supported Mamun et al. (22) from a population-based nationwide study of 10,067 individuals in Bangladesh, which indicated that respondents with chronic diseases were more experienced with depression symptoms during the COVID-19 pandemic. Another study in the same country also reported that participants with chronic diseases had a higher chance of depression and anxiety symptoms during the COVID-19 pandemic (79). During the COVID-19 pandemic, a similar study undertaken in Ireland found that people with chronic conditions had a higher risk of depression, anxiety, and PTSD symptoms (58). Any psychological containment plan should cater to these individuals and provide them with tailored tools and tactics to help them psychologically cope with the COVID-19 crisis. However, in a study of 3,646 Bangladeshi adults aged 18 years or above, Abedin et al. (92) discovered that those with chronic conditions were more likely to be vaccine-hesitant, which is also supported by our findings. Low vaccine understanding, concerns about effectiveness, potential adverse effects, and a lack of trust in vaccines are possible reasons for vaccine refusal. Our findings indicated that people should be health-conscious and vaccinated as soon as possible.

Our findings discovered that unvaccinated populations who were infected with COVID-19 were more likely to suffer from psychological distress, depression, and anxiety symptoms but lower risk of fear symptoms. Consistent with our findings, a recent study conducted in Bangladesh by Abir et al. (93) discovered that those who had been tested for COVID-19 had a higher risk of psychological distress symptoms. Another study in the same country also found the same results (94). A study involving 56,679 adults aged 18 and older from all 34 province-level locations in China found that people with confirmed or suspected COVID-19 had a significantly higher risk of depression and anxiety symptoms, which also matched our findings (57). Moreover, Wang and colleagues (95) observed similar findings in a comprehensive evaluation of 68 research, including 288,830 participants from 19 countries. It's probable that they were worried about the consequences of getting infected with such a dangerous new virus and that they were bored, isolated, and frustrated while in quarantine. However, a study in Bangladesh indicated that those who had previously been infected with COVID-19 were less willing to get vaccinated than those who had not, which is supported by our results (92). This implies a lack of health communication, as there is a widespread misconception that a person gains immunity after recovering from a COVID-19 infection, which may have contributed to this group's unwillingness. The findings of this study point to the necessity for increased public education and risk awareness to take preventive measures to improve COVID-19 pandemic control.

Undoubtedly, unvaccinated populations with poor social support had significantly more significant experience of depression, anxiety, and PTSD symptoms. Our results are consistent with those of Zhang et al. (96), who discovered that people who had less social support had a higher risk of depression and anxiety symptoms. A similar study conducted in China also demonstrated that respondents who had lower social support were more likely to be a chance of depression and PTSD symptoms (56). However, a study conducted in the Philippines found that having more social support was associated with a good intention to obtain the human papillomavirus (HPV) vaccine, which is in line with our findings (97). Social support is vital for dealing with psychological problems and may also be associated with vaccine antibody responses (53, 98). Therefore, the results of this study may guide authorities and policymakers on how to address psychological difficulties and reduce resistance to the COVID-19 vaccine by enlisting the help of family, friends, and coworkers.

## Suggestions

Several actions may and should be taken right now to mitigate the psychological effects of the COVID-19 pandemic on the general public. First, COVID-19 vaccination uptake can be increased after the findings highlighted in this study are addressed, and the immunizations' long-term beneficial and psychological effects are communicated to the general public. To ensure that COVID-19 immunizations reach as many people as possible, the government, public health professionals, and advocates must be prepared to handle vaccine anxiety and boost vaccine education among potential recipients. Evidence-based educational and policy approaches are needed to address these concerns and support COVID-19 vaccination programs. Second, according to the current findings, risky persons should receive special care due to their vulnerability to significant psychological problems. Third, cognitive behavior therapy (CBT) is the most evidence-based treatment, notably internet-based CBT, which can be helpful for mental health interventions during the pandemic. Fourth, the government, non-governmental organizations (NGOs), voluntary organizations, and youth-led projects should offer free tele-counseling and video-counseling, develop psychological support programs for various institutions and workplaces, and develop guidelines for these support services to assist people with mental health problems. Fifth, based on our findings, Bangladesh's general population needs immediate community-based psychosocial support and mental health awareness. Sixth, providing clear communication with regular and transparent updates about the COVID-19 outbreak, advising people to activate their social networks to improve connection with others and maintain their normal daily routine when applicable, and ensuring basic supplies could all help to alleviate mental health problems.

## Strengths and limitations

Some of the study's advantages are as follows: first, this is the first nationwide study in Bangladesh that has evaluated the psychological effects and associated factors among vaccinated and unvaccinated general populations against COVID-19 infection. Second, this groundbreaking study revealed that people's COVID-19 immunization had a significant positive impact on their mental health. Third, it was possible to draw meaningful conclusions from this study because it included all of Bangladesh's divisions and occupations. Fourth, this research will add to our understanding of COVID-19 vaccination and mental health, as well as assist governments and policymakers in developing an effective vaccine campaign to achieve vaccination coverage and herd immunity among various occupational populations during the COVID-19 pandemic. Finally, our findings could be useful in policymaking, identifying high-risk communities, and developing frameworks for population-specific psychological crisis management.

Our research is not without limitations. First, psychological effects were assessed using a self-report technique and an online survey. Only people who have a smartphone and use some SNS/apps participated in the survey. To acquire a more thorough understanding of the situation, future studies should involve clinical interviews or qualitative studies. Second, only the people who had the two doses of vaccine were included in the survey, and those who had one dose of vaccine were excluded. Future studies should examine whether there were comparable differences between those who received a single dose or booster dose of the vaccination and those who did not. Third, because it was a cross-sectional study, there was no way to prove causation. Therefore, this study recommends that longitudinal studies be conducted to overcome this limitation. Fourth, convenient and snowball sampling was used in this study, resulting in selection biases and poor representativeness. Fifth, it is impossible to assess the participation rate because it is unknown how many subjects received the survey link. Sixth, pre-existing co-morbidity (males, Dhaka residents, and unemployed individuals) may also have the effect on mental health following vaccination, which is considered a stressful event. Last but not least, in this study, factors such as which developer's vaccination you received and whether or not you received any vaccine beyond the age of 18 were not considered.

## Conclusion

This study recommends immunizing unvaccinated populations as soon as possible to prevent infection and boost mental health. Males, nuclear family members, married people, daily social media users, people who had COVID-19 infected family members, friends, or colleagues, and people who had lost family members, friends, or colleagues due to the COVID-19 pandemic were associated with higher mental health problems among vaccine recipients. In contrast, participants living in the Dhaka division, having children, unemployed, drinking alcohol, having chronic diseases, being infected with COVID-19, and having poor social support were associated with higher mental health problems among those who did not receive the vaccine. Vulnerable people needed special care, health-related education, and psychological assistance.

## Data availability statement

The original contributions presented in the study are included in the article/Supplementary material, further inquiries can be directed to the corresponding author.

## Ethics statement

The studies involving human participants were reviewed and approved by Department of Psychology, Jagannath University, Dhaka, Bangladesh, and the Ethics Committee of the First Affiliated Hospital, Zhejiang University School of Medicine, Hangzhou, China. The patients/participants provided their written informed consent to participate in this study.

## Author contributions

MDA: conceptualization, methodology, formal analysis, and writing-original draft. MDA, MJA, AI, MM-UH, and OR: data collection. MDA and YX: writing—review and editing. All authors read and approved the final manuscript.

## Funding

This study was supported by the National Natural Science Foundation of China (Grant Nos. 81801340 and 81971271) and the Natural Science Foundation of Zhejiang Province (Grant No. LQ18H090001). The funding sources had no involvement in the study design, data collection, analysis, interpretation of data, writing of the report, or the decision to submit the paper for publication.

## Conflict of interest

The authors declare that the research was conducted in the absence of any commercial or financial relationships that could be construed as a potential conflict of interest.

## Publisher's note

All claims expressed in this article are solely those of the authors and do not necessarily represent those of their affiliated organizations, or those of the publisher, the editors and the reviewers. Any product that may be evaluated in this article, or claim that may be made by its manufacturer, is not guaranteed or endorsed by the publisher.

## References

[1] World Health Organization. WHO Coronavirus (COVID-19) Dashboard. (2022). Available online at: https://www.who.int/publications/m/item/weekly-epidemiological-update-on-covid-19-−22-june-2022 (accessed June 24, 2022).

[2] Ebrahim SH Ahmed QA Gozzer E Schlagenhauf P Memish ZA. Covid-19 and community mitigation strategies in a pandemic. BMJ. (2020) 368:m1066. 10.1136/bmj.m1066 32184233

[3] Chen S Yang J Yang W Wang C Bärnighausen T. COVID-19 control in China during mass population movements at new year. Lancet. (2020) 395:764–6. 10.1016/S0140-6736(20)30421-9 32105609 PMC7159085

[4] Adalja AA Toner E Inglesby TV. Priorities for the US Health Community Responding to COVID-19. JAMA. (2020) 323:1343–4. 10.1001/jama.2020.3413 32125355

[5] Bao Y Sun Y Meng S Shi J Lu L. 2019-nCoV epidemic: address mental health care to empower society. Lancet. (2020) 395:e37–8. 10.1016/S0140-6736(20)30309-3 32043982 PMC7133594

[6] Mak IW Chu CM Pan PC Yiu MG Chan VL. Long-term psychiatric morbidities among SARS survivors. Gen Hosp Psychiatry. (2009) 31:318–26. 10.1016/j.genhosppsych.2009.03.001 19555791 PMC7112501

[7] Lee AM Wong JG McAlonan GM Cheung V Cheung C Sham PC. Stress and psychological distress among SARS survivors 1 year after the outbreak. Can J Psychiatry. (2007) 52:233–40. 10.1177/070674370705200405 17500304

[8] Jalloh MF Li W Bunnell RE Ethier KA O'Leary A Hageman KM. Impact of Ebola experiences and risk perceptions on mental health in Sierra Leone, July 2015. BMJ Glob Health. (2018) 3:e000471. 10.1136/bmjgh-2017-000471 29607096 PMC5873549

[9] Jeong H Yim HW Song Y-J Ki M Min J-A Cho J. Mental health status of people isolated due to Middle East Respiratory Syndrome. Epidemiol Health. (2016) 38:e2016048. 10.4178/epih.e2016048 28196409 PMC5177805

[10] Chau SWH Wong OWH Ramakrishnan R Chan SSM Wong EKY Li PYT. History for some or lesson for all? A systematic review and meta-analysis on the immediate and long-term mental health impact of the 2002-2003 Severe Acute Respiratory Syndrome (SARS) outbreak. BMC Public Health. (2021) 21:670. 10.1186/s12889-021-10701-3 33827499 PMC8025448

[11] Pan KY Kok AAL Eikelenboom M Horsfall M Jörg F Luteijn RA. The mental health impact of the COVID-19 pandemic on people with and without depressive, anxiety, or obsessive-compulsive disorders: a longitudinal study of three Dutch case-control cohorts. Lancet Psychiatry. (2021) 8:121–9. 10.1016/S2215-0366(20)30491-0 33306975 PMC7831806

[12] Xiong J Lipsitz O Nasri F Lui LMW Gill H Phan L. Impact of COVID-19 pandemic on mental health in the general population: a systematic review. J Affect Disord. (2020) 277:55–64. 10.1016/j.jad.2020.08.001 32799105 PMC7413844

[13] Nochaiwong S Ruengorn C Thavorn K Hutton B Awiphan R Phosuya C. Global prevalence of mental health issues among the general population during the coronavirus disease-2019 pandemic: a systematic review and meta-analysis. Sci Rep. (2021) 11:10173. 10.1038/s41598-021-89700-8 33986414 PMC8119461

[14] Zhao YJ Jin Y Rao WW Li W Zhao N Cheung T. The prevalence of psychiatric comorbidities during the SARS and COVID-19 epidemics: a systematic review and meta-analysis of observational studies. J Affect Disord. (2021) 287:145–57. 10.1016/j.jad.2021.03.016 33799032 PMC7948672

[15] Islam SMD Bodrud-Doza M Khan RM Haque MA Mamun MA. Exploring COVID-19 stress and its factors in Bangladesh: a perception-based study. Heliyon. (2020) 6:e04399. 10.1016/j.heliyon.2020.e04399 32685726 PMC7350787

[16] Institute Institute of Epidemiology Disease Control Research (IEDCR). Covid-19 Status Bangladesh. (2020). Available online at: https://iedcr.gov.bd/covid-19/ (accessed January 13, 2022).

[17] Anwar S Nasrullah M Hosen MJ. COVID-19 and Bangladesh: challenges and how to address them. Front Public Health. (2020) 8:154. 10.3389/fpubh.2020.00154 32426318 PMC7203732

[18] The Daily Star. Coronavirus Outbreak: Govt Orders Closure of Public, Private Offices from March 26 to April 4. (2020). Available online at: https://www.thedailystar.net/coronavirus-deadly-new-threat/news/govt-offices-closed-march-26-april-4-cabinet-secretary-1884730 (accessed January 15, 2022).

[19] The Daily Star. Coronavirus Outbreak: Shutdown Won't Be Extended after May 30. (2020). Available online at: https://www.thedailystar.net/coronavirusoutbreak-shutdown-wont-be-extended-after-may-30-1905826 (accessed January 15, 2022).

[20] Dhaka Tribune,. Restriction on Public Movement Extended till August 31. (2020). Available online at: https://www.dhakatribune.com/health/coronavirus/2020/08/03/restriction-on-public-movement-extended-till-august-31 (accessed January 15, 2022).

[21] Zubayer AA Rahman ME Islam MB Babu SZD Rahman QM Bhuiyan M. Psychological states of Bangladeshi people four months after the COVID-19 pandemic: an online survey. Heliyon. (2020) 6:e05057. 10.1016/j.heliyon.2020.e05057 33015396 PMC7521899

[22] Mamun MA Sakib N Gozal D Bhuiyan AI Hossain S Bodrud-Doza M. The COVID-19 pandemic and serious psychological consequences in Bangladesh: a population-based nationwide study. J Affect Disord. (2021) 279:462–72. 10.1016/j.jad.2020.10.036 33120247 PMC7568472

[23] Islam MS Ferdous MZ Potenza MN. Panic and generalized anxiety during the COVID-19 pandemic among Bangladeshi people: an online pilot survey early in the outbreak. J Affect Disord. (2020) 276:30–7. 10.1016/j.jad.2020.06.049 32697713 PMC7362838

[24] Banna MHA Sayeed A Kundu S Christopher E Hasan MT Begum MR. The impact of the COVID-19 pandemic on the mental health of the adult population in Bangladesh: a nationwide cross-sectional study. Int J Environ Health Res. (2022) 32:850–61. 10.1080/09603123.2020.1802409 32741205

[25] Dubé E Laberge C Guay M Bramadat P Roy R Bettinger J. Vaccine hesitancy: an overview. Hum Vaccin Immunother. (2013) 9:1763–73. 10.4161/hv.24657 23584253 PMC3906279

[26] Larson HJ Jarrett C Schulz WS Chaudhuri M Zhou Y Dube E. Measuring vaccine hesitancy: the development of a survey tool. Vaccine. (2015) 33:4165–75. 10.1016/j.vaccine.2015.04.037 25896384

[27] MacDonald NE. Vaccine hesitancy: definition, scope and determinants. Vaccine. (2015) 33:4161–4. 10.1016/j.vaccine.2015.04.036 25896383

[28] Sanche S Lin YT Xu C Romero-Severson E Hengartner N Ke R. High contagiousness and rapid spread of severe acute respiratory syndrome coronavirus 2. Emerg Infect Dis. (2020) 26:1470–7. 10.3201/eid2607.200282 32255761 PMC7323562

[29] World Health Organization. Ten Threats to Global Health in 2019. (2019). Available online at: https://www.who.int/newsroom/spotlight/ten-threats-to-global-health-in-2019 (accessed January 18, 2022).

[30] World Health Organization. WHO Bangladesh COVID-19 Morbidity and Mortality Weekly Update (MMWU). (2022). Available online at: https://cdn.who.int/media/docs/default-source/searo/bangladesh/covid-19-who-bangladesh-situation-reports/who_ban_sitrep_121_20220620.pdf?sfvrsn=5142caa8_1 (accessed June 24, 2022).

[31] Directorate General of Health Services. COVID-19 Dynamic Dashboard for Bangladesh. (2022). Available online at: https://dghs-dashboard.com/pages/covid19.php (accessed June 24, 2022).

[32] Allergic Allergic reactions including anaphylaxis after receipt of the first dose of Pfizer-BioNTech COVID-19 vaccine - United States December 14-23 2020. MMWR Morb Mortal Wkly Rep. (2021) 70:46–51. 10.15585/mmwr.mm7002e1 33444297 PMC7808711

[33] Allergic Allergic reactions including anaphylaxis after receipt of the first dose of moderna COVID-19 vaccine - United States December 21 2020-January 10 2021. MMWR Morb Mortal Wkly Rep. (2021) 70:125–9. 10.15585/mmwr.mm7004e1 33507892 PMC7842812

[34] Kaplan RM Milstein A. Influence of a COVID-19 vaccine's effectiveness and safety profile on vaccination acceptance. Proc Natl Acad Sci USA. (2021) 118:e2021726118. 10.1073/pnas.2021726118 33619178 PMC7958192

[35] Perez-Arce F Angrisani M Bennett D Darling J Kapteyn A Thomas K. COVID-19 vaccines and mental distress. PLoS ONE. (2021) 16:e0256406. 10.1371/journal.pone.0256406 34496006 PMC8425550

[36] Zheng YB Sun J Liu L Zhao YM Yan W Yuan K. COVID-19 vaccine-related psychological stress among general public in China. Front Psychiatry. (2021) 12:774504. 10.3389/fpsyt.2021.774504 34950070 PMC8689133

[37] Bendau A Plag J Petzold MB Ströhle A. COVID-19 vaccine hesitancy and related fears and anxiety. Int Immunopharmacol. (2021) 97:107724. 10.1016/j.intimp.2021.107724 33951558 PMC8078903

[38] Al-Amer R Malak MZ Burqan HMR Stănculescu E Nalubega S Alkhamees AA. Emotional reaction to the first dose of COVID-19 vaccine: postvaccination decline in anxiety and stress among anxious individuals and increase among individuals with normal prevaccination anxiety levels. J Pers Med. (2022) 12:912. 10.3390/jpm12060912 35743695 PMC9224616

[39] Goldberg DP Gater R Sartorius N Ustun TB Piccinelli M Gureje O. The validity of two versions of the GHQ in the WHO study of mental illness in general health care. Psychol Med. (1997) 27:191–7. 10.1017/S0033291796004242 9122299

[40] Islam MN Iqbal K. Mental health and social support. Chittagong Univ J Biol Sci. (2008) 3:95–107. 10.3329/cujbs.v3i1.13410

[41] Kroenke K Spitzer RL Williams JB. The Patient Health Questionnaire-2: validity of a two-item depression screener. Med Care. (2003) 41:1284–92. 10.1097/01.MLR.0000093487.78664.3C 14583691

[42] Chowdhury AN Ghosh S Sanyal D. Bengali adaptation of brief patient health questionnaire for screening depression at primary care. J Indian Med Assoc. (2004) 102:544–7. Available online at: https://www.researchgate.net/publication/7853095_Bengali_adaptation_of_Brief_Patient_Health_Questionnaire_for_screening_depression_at_primary_care (accessed January 25, 2022). 15887819

[43] Kroenke K Spitzer RL Williams JB Monahan PO Löwe B. Anxiety disorders in primary care: prevalence, impairment, comorbidity, and detection. Ann Intern Med. (2007) 146:317–25. 10.7326/0003-4819-146-5-200703060-00004 17339617

[44] Haque M Das C Ara R Alam M Ullah S Hossain Z. Prevalence of generalized anxiety disorder and its effect on daily living in the rural community of Rajshahi. TAJ: J. Teach. Assoc. (2014) 27:14–23. 10.3329/taj.v27i1.37603

[45] Cohen S Kamarck T Mermelstein R. A global measure of perceived stress. J Health Soc Behav. (1983) :385-96. 10.2307/2136404 6668417

[46] Mozumder MK. Validation of Bengali perceived stress scale among LGBT population. BMC Psychiatry. (2017) 17:1–7. 10.1186/s12888-017-1482-0 28851332 PMC5576282

[47] Prins A Bovin MJ Smolenski DJ Marx BP Kimerling R Jenkins-Guarnieri MA. The primary care PTSD screen for DSM-5 (PC-PTSD-5): development and evaluation within a veteran primary care sample. J Gen Intern Med. (2016) 31:1206–11. 10.1007/s11606-016-3703-5 27170304 PMC5023594

[48] Alam MD PS Momi M Ni L Xu Y. Factors associated with psychological outcomes among vaccinated and unvaccinated health care workers against COVID-19 infection in Bangladesh. Front. Med. (2022) 9:852922. 10.3389/fmed.2022.852922 35402432 PMC8988188

[49] Bastien CH Vallières A Morin CM. Validation of the Insomnia Severity Index as an outcome measure for insomnia research. Sleep Med. (2001) 2:297–307. 10.1016/S1389-9457(00)00065-4 11438246

[50] Mamun MA Alimoradi Z Gozal D Manzar MD Broström A Lin C-Y. Validating Insomnia Severity Index (ISI) in a Bangladeshi population: using classical test theory and rasch analysis. Int J Environ Res Public Health. (2022) 19:225. 10.3390/ijerph19010225 35010485 PMC8750940

[51] Ahorsu DK Lin CY Imani V Saffari M Griffiths MD Pakpour AH. The fear of COVID-19 scale: development and initial validation. Int J Ment Health Addict. (2020) 20:1537–45. 10.1007/s11469-020-00270-8 32226353 PMC7100496

[52] Sakib N Bhuiyan A Hossain S Al Mamun F Hosen I Abdullah AH. Psychometric validation of the bangla fear of COVID-19 scale: confirmatory factor analysis and rasch analysis. Int J Ment Health Addict. (2020) 11:1–12. 10.1007/s11469-020-00289-x 32395096 PMC7213549

[53] Kocalevent R-D Berg L Beutel ME Hinz A Zenger M Härter M. Social support in the general population: standardization of the Oslo social support scale (OSSS-3). BMC Psychol. (2018) 6:1–8. 10.1186/s40359-018-0249-9 30016997 PMC6050647

[54] Koltai J Raifman J Bor J McKee M Stuckler D. Does COVID-19 vaccination improve mental health? A difference-in-difference analysis of the Understanding Coronavirus in America study. medRxiv. [Preprint]. (2021). 10.1101/2021.07.19.21260782 35012830 PMC8674498

[55] Bilge Y Keles E Baydili KN. The impact of COVID-19 vaccination on mental health. J Loss Trauma. (2021) 27:285–88. 10.1080/15325024.2021.196355833552172

[56] Song X Fu W Liu X Luo Z Wang R Zhou N. Mental health status of medical staff in emergency departments during the Coronavirus disease 2019 epidemic in China. Brain Behav Immun. (2020) 88:60–5. 10.1016/j.bbi.2020.06.002 32512134 PMC7273140

[57] Shi L Lu ZA Que JY Huang XL Liu L Ran MS. Prevalence of and risk factors associated with mental health symptoms among the general population in China during the coronavirus disease 2019 pandemic. JAMA Netw Open. (2020) 3:e2014053. 10.1001/jamanetworkopen.2020.14053 32609353 PMC7330717

[58] Karatzias T Shevlin M Murphy J McBride O Ben-Ezra M Bentall RP. Posttraumatic stress symptoms and associated comorbidity during the COVID-19 pandemic in Ireland: a population-based study. J Trauma Stress. (2020) 33:365–70. 10.1002/jts.22565 32662129 PMC7405473

[59] Zhong BL Luo W Li HM Zhang QQ Liu XG Li WT. Knowledge, attitudes, and practices towards COVID-19 among Chinese residents during the rapid rise period of the COVID-19 outbreak: a quick online cross-sectional survey. Int J Biol Sci. (2020) 16:1745–52. 10.7150/ijbs.45221 32226294 PMC7098034

[60] Li LQ Huang T Wang YQ Wang ZP Liang Y Huang TB. COVID-19 patients' clinical characteristics, discharge rate, and fatality rate of meta-analysis. J Med Virol. (2020) 92:577–83. 10.1002/jmv.25757 32162702 PMC7228329

[61] Banik R Islam MS Pranta MUR Rahman QM Rahman M Pardhan S. Understanding the determinants of COVID-19 vaccination intention and willingness to pay: findings from a population-based survey in Bangladesh. BMC Infect Dis. (2021) 21:892. 10.1186/s12879-021-06406-y 34465297 PMC8406014

[62] Paul A Sikdar D Mahanta J Ghosh S Jabed MA Paul S. Peoples' understanding, acceptance, and perceived challenges of vaccination against COVID-19: A cross-sectional study in Bangladesh. PLoS ONE. (2021) 16:e0256493. 10.1371/journal.pone.0256493 34415969 PMC8378750

[63] Mehareen J Rahman MA Dhira TA Sarker AR. Prevalence and socio-demographic correlates of depression, anxiety, and co-morbidity during COVID-19: a cross-sectional study among public and private university students of Bangladesh. J Affect Disord Rep. (2021) 5:100179. 10.1016/j.jadr.2021.100179

[64] Islam MS Siddique AB Akter R Tasnim R Sujan MSH Ward PR. Knowledge, attitudes and perceptions towards COVID-19 vaccinations: a cross-sectional community survey in Bangladesh. BMC Public Health. (2021) 21:1851. 10.1186/s12889-021-11880-9 34645399 PMC8513387

[65] Yang Y Dobalian A Ward KD. COVID-19 vaccine hesitancy and its determinants among adults with a history of tobacco or marijuana use. J Community Health. (2021) 46:1090–8. 10.1007/s10900-021-00993-2 33956270 PMC8101333

[66] Ghaleb Y Lami F Al Nsour M Rashak HA Samy S Khader YS. Mental health impacts of COVID-19 on healthcare workers in the Eastern Mediterranean Region: a multi-country study. J Public Health. (2021) 43 (Suppl. 3):iii34–iii42. 10.1093/pubmed/fdab321 34642765 PMC8524602

[67] Debnath PR Islam MS Karmakar PK Sarker R Zhai ZW Potenza MN. Mental health concerns, insomnia, and loneliness among intern doctors amidst the COVID-19 pandemic: evidence from a large tertiary care hospital in Bangladesh. Int J Ment Health Addict. (2021) 11:1–21. 10.1007/s11469-021-00690-0 34840536 PMC8604201

[68] Rahman MM Chisty MA Sakib MS Quader MA Shobuj IA Alam MA. Status and perception toward the COVID-19 vaccine: a cross-sectional online survey among adult population of Bangladesh. Health Sci Rep. (2021) 4:e451. 10.1002/hsr2.451 34938896 PMC8671900

[69] Wang J Jing R Lai X Zhang H Lyu Y Knoll MD. Acceptance of COVID-19 vaccination during the COVID-19 pandemic in China. Vaccines. (2020) 8:482. 10.3390/vaccines8030482 32867224 PMC7565574

[70] Hasan MT Hossain S Safa F Anjum A Khan AH Koly KN. Prevalence of anxiety and depressive symptoms among physicians during the COVID-19 pandemic in Bangladesh: a cross-sectional study. medRxiv. [Preprint]. (2020). 10.1101/2020.12.08.20245829 36606239 PMC9253439

[71] Hossain MT Ahammed B Chanda SK Jahan N Ela MZ Islam MN. Social and electronic media exposure and generalized anxiety disorder among people during COVID-19 outbreak in Bangladesh: a preliminary observation. PLoS ONE. (2020) 15:e0238974. 10.1371/journal.pone.0238974 32916691 PMC7486135

[72] Adebisi YA Alaran AJ Bolarinwa OA Akande-Sholabi W Lucero-Prisno DE. When it is available, will we take it? Social media users' perception of hypothetical COVID-19 vaccine in Nigeria. Pan Afr Med J. (2021) 38:230. 10.11604/pamj.2021.38.230.27325 34046135 PMC8140724

[73] Piltch-Loeb R Savoia E Goldberg B Hughes B Verhey T Kayyem J. Examining the effect of information channel on COVID-19 vaccine acceptance. PLoS ONE. (2021) 16:e0251095. 10.1371/journal.pone.0251095 33979370 PMC8116041

[74] González-Sanguino C Ausín B Castellanos M Saiz J López-Gómez A Ugidos C. Mental health consequences during the initial stage of the 2020 coronavirus pandemic (COVID-19) in Spain. Brain Behav Immun. (2020) 87:172–6. 10.1016/j.bbi.2020.05.040 32405150 PMC7219372

[75] Akiful Haque MM Rahman ML Hossian M Matin KF Nabi MH Saha S. Acceptance of COVID-19 vaccine and its determinants: evidence from a large sample study in Bangladesh. Heliyon. (2021) 7:e07376. 10.1016/j.heliyon.2021.e07376 34189332 PMC8223020

[76] Moghanibashi-Mansourieh A. Assessing the anxiety level of Iranian general population during COVID-19 outbreak. Asian J Psychiatr. (2020) 51:102076. 10.1016/j.ajp.2020.102076 32334409 PMC7165107

[77] Tahir MJ Saqlain M Tariq W Waheed S Tan SHS Nasir SI. Population preferences and attitudes towards COVID-19 vaccination: a cross-sectional study from Pakistan. BMC Public Health. (2021) 21:1759. 10.1186/s12889-021-11814-5 34565351 PMC8474768

[78] Abir T Kalimullah NA Osuagwu UL Nur AYDM Husain T Goson PC. Prevalence and factors associated with mental health impact of COVID-19 pandemic in Bangladesh: a survey-based cross-sectional study. Ann Glob Health. (2021) 87:43. 10.5334/aogh.3269 33981590 PMC8086734

[79] Tasnim R Sujan MSH Islam MS Ferdous MZ Hasan MM Koly KN. Depression and anxiety among individuals with medical conditions during the COVID-19 pandemic: findings from a nationwide survey in Bangladesh. Acta Psychol. (2021) 220:103426. 10.1016/j.actpsy.2021.103426 34619554 PMC8486640

[80] Ferdous MZ Islam MS Sikder MT Mosaddek ASM Zegarra-Valdivia JA Gozal D. Knowledge, attitude, and practice regarding COVID-19 outbreak in Bangladesh: an online-based cross-sectional study. PLoS ONE. (2020) 15:e0239254. 10.1371/journal.pone.0239254 33035219 PMC7546509

[81] Islam S Emran GI Rahman E Banik R Sikder T Smith L. Knowledge, attitudes and practices associated with the COVID-19 among slum dwellers resided in Dhaka City: a Bangladeshi interview-based survey. J Public Health. (2021) 43:13–25. 10.1093/pubmed/fdaa182 33057666 PMC7665690

[82] Santamaría MD Mondragon NI Santxo NB Ozamiz-Etxebarria N. Teacher stress, anxiety and depression at the beginning of the academic year during the COVID-19 pandemic. Glob Ment Health. (2021) 8:e14. 10.1017/gmh.2021.14 34192001 PMC8082122

[83] Khubchandani J Sharma S Price JH Wiblishauser MJ Sharma M Webb FJ. COVID-19 vaccination hesitancy in the United States: a rapid national assessment. J Community Health. (2021) 46:270–7. 10.1007/s10900-020-00958-x 33389421 PMC7778842

[84] Khan MAS Debnath S Islam MS Zaman S Ambia NE Barshan AD. Mental health of young people amidst COVID-19 pandemic in Bangladesh. Heliyon. (2021) 7:e07173. 10.1016/j.heliyon.2021.e07173 34075348 PMC8161733

[85] Das R Hasan MR Daria S Islam MR. Impact of COVID-19 pandemic on mental health among general Bangladeshi population: a cross-sectional study. BMJ Open. (2021) 11:e045727. 10.1136/bmjopen-2020-045727 33837107 PMC8042595

[86] Ali M Hossain A. What is the extent of COVID-19 vaccine hesitancy in Bangladesh? A cross-sectional rapid national survey. BMJ Open. (2021) 11:e050303. 10.1136/bmjopen-2021-050303 34429316 PMC8387740

[87] Dror AA Eisenbach N Taiber S Morozov NG Mizrachi M Zigron A. Vaccine hesitancy: the next challenge in the fight against COVID-19. Eur J Epidemiol. (2020) 35:775–9. 10.1007/s10654-020-00671-y 32785815 PMC8851308

[88] Tasnim R Sujan MSH Islam MS Ritu AH Siddique MAB Toma TY. Prevalence and correlates of anxiety and depression in frontline healthcare workers treating people with COVID-19 in Bangladesh. BMC Psychiatry. (2021) 21:271. 10.1186/s12888-021-03243-w 34034679 PMC8146174

[89] Mental Health Foundation. Alcohol and Mental Health. (2022). Available online at: https://www.mentalhealth.org.uk/a-to-z/a/alcohol-and-mental-health#:~:text=In%20the%20long%2Dterm%2C%20alcohol,start%20a%20cycle%20of%20dependence (accessed June 11, 2022).

[90] Okubo R Yoshioka T Ohfuji S Matsuo T Tabuchi T. COVID-19 vaccine hesitancy and its associated factors in Japan. Vaccines. (2021) 9:662. 10.3390/vaccines9060662 34204465 PMC8234307

[91] Gusar I Konjevoda S Babić G Hnatešen D Cebohin M Orlandini R. Pre-vaccination COVID-19 vaccine literacy in a croatian adult population: a cross-sectional study. Int J Environ Res Public Health. (2021) 18:7073. 10.3390/ijerph18137073 34281009 PMC8297136

[92] Abedin M Islam MA Rahman FN Reza HM Hossain MZ Hossain MA. Willingness to vaccinate against COVID-19 among Bangladeshi adults: understanding the strategies to optimize vaccination coverage. PLoS ONE. (2021) 16:e0250495. 10.1371/journal.pone.0250495 33905442 PMC8078802

[93] Abir T Osuagwu UL Kalimullah NA Yazdani DMN Husain T Basak P. Psychological impact of COVID-19 pandemic in Bangladesh: analysis of a cross-sectional survey. Health Secur. (2021) 19:468–78. 10.1089/hs.2020.0205 34348050

[94] Sabrina F Chowdhury MTH Nath SK Imon AA Quader SMA Jahan MS. Psychological distress among bangladeshi dental students during the COVID-19 pandemic. Int J Environ Res Public Health. (2021) 19:176. 10.3390/ijerph19010176 35010435 PMC8750223

[95] Wang Y Kala MP Jafar TH. Factors associated with psychological distress during the coronavirus disease 2019 (COVID-19) pandemic on the predominantly general population: a systematic review and meta-analysis. PLoS ONE. (2020) 15:e0244630. 10.1371/journal.pone.0244630 33370404 PMC7769562

[96] Zhang C Yang L Liu S Ma S Wang Y Cai Z. Survey of insomnia and related social psychological factors among medical staff involved in the 2019 novel coronavirus disease outbreak. Front Psychiatry. (2020) 11:306. 10.3389/fpsyt.2020.00306 32346373 PMC7171048

[97] Young AM Crosby RA Jagger KS Richardson MB Kloha RA Safarian V. HPV vaccine acceptability among women in the Philippines. Asian Pac J Cancer Prev. (2010) 11:1781–7. Available online at: http://journal.waocp.org/article_25450_954d0fcbc1f01fc9238d667e8fbd6192.pdf (accessed January 29, 2022). 21338233

[98] Uchino BN Landvatter J Zee K Bolger N. Social support and antibody responses to vaccination: a meta-analysis. Ann Behav Med. (2020) 54:567–74. 10.1093/abm/kaaa029 32415849 PMC7414291

