# Supplementary material for: Psychological effects and associated factors among vaccinated and unvaccinated general population against COVID-19 infection in Bangladesh
Source: Front Psychiatry. 2022 Aug 12;13:916160. doi: 10.3389/fpsyt.2022.916160 (PMC9412971; doi:10.3389/fpsyt.2022.916160)
Supplement: Supplementary file 2 [file Table_2.docx]

**Supplement Table S2.** Multivariate logistic regression analysis of factors associated with psychological effects among vaccinated and unvaccinated populations against COVID-19 infection.

| **Psychological effects** | **Sex** | | | |  | **Age, y** | | | | | | | | | |  | **Divisions of Bangladesh** | | | | | | | | | | | | | | | |
| --- | --- | --- | --- | --- | --- | --- | --- | --- | --- | --- | --- | --- | --- | --- | --- | --- | --- | --- | --- | --- | --- | --- | --- | --- | --- | --- | --- | --- | --- | --- | --- | --- |
|  | **Male** | | **Female** | |  | **18-29** | | **30-39** | | **40-49** | | **50-59** | | **≥60** | |  | **Dhaka** | | **Chittagong** | | **Barisal** | | **Khulna** | | **Rajshahi** | | **Rangpur** | | **Mymensingh** | | **Sylhet** | |
|  | **AOR**  **(95% CI)** | ***p* value** | **AOR**  **(95% CI)** | ***p***  **value** |  | **AOR**  **(95% CI)** | ***p* value** | **AOR**  **(95% CI)** | ***p***  **value** | **AOR**  **(95% CI)** | ***p* value** | **AOR**  **(95% CI)** | ***p***  **value** | **AOR**  **(95% CI)** | ***p* value** |  | **AOR**  **(95% CI)** | ***p* value** | **AOR**  **(95% CI)** | ***p***  **value** | **AOR**  **(95% CI)** | ***p* value** | **AOR**  **(95% CI)** | ***p* value** | **AOR**  **(95% CI)** | ***p***  **value** | **AOR**  **(95% CI)** | ***p* value** | **AOR**  **(95% CI)** | ***p***  **value** | **AOR**  **(95% CI)** | ***p***  **value** |
| **Psychological distress symptoms** | | | | | | | | | | | | | | | | | | | | | | | | | | | | | | | | |
| Total | 1.07  (0.91-1.25) | 0.41 | 1  [Reference] |  |  | 0.90  (0.40-2.00) | 0.80 | 0.76  (0.35-1.65) | 0.49 | 0.97  (0.44-2.12) | 0.94 | 1.39  (0.57-3.39) | 0.46 | 1  [Reference] |  |  | 1.14  (0.82-1.59) | 0.42 | 1.18  (0.81-1.70) | 0.37 | 1.01  (0.68-1.49) | 0.94 | 1.07  (0.73-1.55) | 0.71 | 1.21  (0.79-1.85) | 0.37 | 1.00  (0.66-1.52) | 0.96 | 1.32  (0.81-2.17) | 0.25 | 1  [Reference] |  |
| Vaccinated  population | 0.98  (0.76-1.27) | 0.91 | 1  [Reference] |  |  | 2.23  (0.64-7.71) | 0.20 | 1.51  (0.45-5.00) | 0.49 | 1.94  (0.58-6.47) | 0.28 | 2.55  (0.66-9.83) | 0.17 | 1  [Reference] |  |  | 1.03  (0.57-1.87) | 0.90 | 1.25  (0.64-2.44) | 0.50 | 0.89  (0.42-1.87) | 0.76 | 1.18  (0.59-2.33) | 0.62 | 1.24  (0.53-2.90) | 0.61 | 1.07  (0.48-2.42) | 0.85 | 8.68  (1.79-41.9) | 0.00 | 1  [Reference] |  |
| Unvaccinated population | 1.14  (0.91-1.42) | 0.23 | 1  [Reference] |  |  | 0.46  (0.15-1.34) | 0.15 | 0.43  (0.15-1.22) | 0.11 | 0.58  (0.19-1.61) | 0.28 | 0.85  (0.25-2.90) | 0.80 | 1  [Reference] |  |  | 1.37  (0.90-2.08) | 0.13 | 1.18  (0.75-1.86) | 0.45 | 1.09  (0.68-1.75) | 0.70 | 1.05  (0.66-1.67) | 0.82 | 1.28  (0.77-2.12) | 0.33 | 1.05  (0.64-1.73) | 0.83 | 1.04  (0.58-1.85) | 0.88 | 1  [Reference] |  |
| **Depression symptoms** | | |  | | | | | | | | | | | | | | | | | | | | | | | | | | | | | |
| Total | 1.78  (1.16-1.93) | 0.00 | 1  [Reference] |  |  | 0.59  (0.23-1.47) | 0.26 | 0.58  (0.24-1.44) | 0.25 | 0.54  (0.22-1.33) | 0.18 | 0.45  (0.16-1.20) | 0.11 | 1  [Reference] |  |  | 1.39  (1.11-1.96) | 0.05 | 1.12  (0.76-1.63) | 0.55 | 1.11  (0.74-1.65) | 0.60 | 0.79  (0.54-1.16) | 0.23 | 1.16  (0.75-1.80) | 0.49 | 0.78  (0.51-1.19) | 0.24 | 0.99  (0.60-1.63) | 0.97 | 1  [Reference] |  |
| Vaccinated  population | 1.80  (1.14-2.14) | 0.00 | 1  [Reference] |  |  | 0.67  (0.12-3.54) | 0.63 | 0.87  (0.17-4.44) | 0.87 | 0.95  (0.18-4.89) | 0.95 | 0.57  (0.10-3.24) | 0.53 | 1  [Reference] |  |  | 2.40  (1.29-4.45) | 0.00 | 1.23  (0.62-2.43) | 0.55 | 1.17  (0.55-2.51) | 0.67 | 1.19  (0.59-2.40) | 0.61 | 1.88  (0.76-4.63) | 0.16 | 0.78  (0.35-1.74) | 0.54 | 0.49  (0.17-1.34) | 0.16 | 1  [Reference] |  |
| Unvaccinated population | 1.23  (0.66-1.63) | 0.09 | 1  [Reference] |  |  | 0.53  (0.17-1.62) | 0.27 | 0.51  (0.17-1.50) | 0.22 | 0.40  (0.13-1.18) | 0.09 | 0.40  (0.11-1.39) | 0.15 | 1  [Reference] |  |  | 2.06  (1.40-2.52) | 0.00 | 0.99  (0.62-1.57) | 0.97 | 0.94  (0.59-1.52) | 0.82 | 0.60  (0.37-0.95) | 0.03 | 0.91  (0.54-1.52) | 0.73 | 0.73  (0.45-1.20) | 0.22 | 1.04  (0.58-1.87) | 0.88 | 1  [Reference] |  |
| **Anxiety symptoms** | | | | |  |  | | | | | | | | | | | | | | | | | | | | | | | | | | |
| Total | 1.71  (1.21-2.05) | 0.00 | 1  [Reference] |  |  | 0.48  (0.19-1.23) | 0.13 | 0.56  (0.22-1.41) | 0.22 | 0.71  (0.28-1.78) | 0.47 | 0.63  (0.23-1.76) | 0.38 | 1  [Reference] |  |  | 0.73  (0.51-1.03) | 0.07 | 1.02  (0.69-1.51) | 0.89 | 1.19  (0.79-1.80) | 0.39 | 0.89  (0.60-1.31) | 0.56 | 0.89  (0.57-1.39) | 0.62 | 0.81  (0.53-1.25) | 0.35 | 0.80  (0.48-1.34) | 0.41 | 1  [Reference] |  |
| Vaccinated  population | 1.79  (1.13-2.04) | 0.04 | 1  [Reference] |  |  | 0.27  (0.03-2.38) | 0.24 | 0.33  (0.03-2.77) | 0.30 | 0.50  (0.05-4.27) | 0.52 | 0.27  (0.03-2.80) | 0.29 | 1  [Reference] |  |  | 1.83  (0.96-3.48) | 0.06 | 1.25  (0.61-2.57) | 0.53 | 1.85  (0.81-4.25) | 0.14 | 1.10  (0.53-2.27) | 0.79 | 1.31  (0.54-3.21) | 0.54 | 0.61  (0.26-1.41) | 0.25 | 0.60  (0.20-1.78) | 0.34 | 1  [Reference] |  |
| Unvaccinated population | 1.61  (1.01-1.93) | 0.03 | 1  [Reference] |  |  | 0.79  (0.26-2.38) | 0.67 | 0.89  (0.30-2.61) | 0.83 | 0.91  (0.31-2.71) | 0.87 | 1.08  (0.31-3.76) | 0.89 | 1  [Reference] |  |  | 1.86  (1.15-2.47) | 0.00 | 0.90  (0.57-1.43) | 0.67 | 0.93  (0.58-1.51) | 0.79 | 0.78  (0.49-1.25) | 0.30 | 0.74  (0.44-1.24) | 0.26 | 0.90  (0.55-1.50) | 0.71 | 0.70  (0.39-1.26) | 0.24 | 1  [Reference] |  |
| **Stress symptoms** | | | | |  |  | | | | | | | | | | | | | | | | | | | | | | | | | | |
| Total | 1.08  (0.90-1.29) | 0.36 | 1  [Reference] |  |  | 1.45  (0.62-3.36) | 0.38 | 1.49  (0.66-3.37) | 0.33 | 1.31  (0.57-2.99) | 0.51 | 1.05  (0.42-2.62) | 0.91 | 1  [Reference] |  |  | 1.25  (0.87-1.79) | 0.21 | 1.11  (0.74-1.65) | 0.59 | 0.99  (0.65-1.50) | 0.96 | 1.24  (0.82-1.87) | 0.29 | 0.85  (0.54-1.34) | 0.50 | 1.06  (0.68-1.66) | 0.78 | 0.56  (0.34-0.92) | 0.04 | 1  [Reference] |  |
| Vaccinated  population | 1.29  (0.93-1.72) | 0.13 | 1  [Reference] |  |  | 1.39  (0.33-5.79) | 0.64 | 2.05  (0.51-8.14) | 0.30 | 1.38  (0.34-5.54) | 0.64 | 1.48  (0.32-6.75) | 0.60 | 1  [Reference] |  |  | 1.54  (0.79-3.01) | 0.20 | 0.94  (0.45-1.97) | 0.87 | 1.97  (0.81-4.77) | 0.13 | 1.41  (0.65-3.07) | 0.37 | 1.99  (0.72-5.48) | 0.18 | 2.81  (0.99-7.94) | 0.05 | 0.41  (0.14-1.16) | 0.09 | 1  [Reference] |  |
| Unvaccinated population | 1.03  (0.82-1.30) | 0.76 | 1  [Reference] |  |  | 1.55  (0.54-4.44) | 0.40 | 1.34  (0.48-3.71) | 0.56 | 1.40  (0.50-3.90) | 0.51 | 0.94  (0.28-3.06) | 0.91 | 1  [Reference] |  |  | 1.92  (1.12-2.88) | 0.00 | 1.24  (0.77-2.01) | 0.37 | 0.81  (0.50-1.32) | 0.41 | 1.17  (0.71-1.90) | 0.52 | 0.71  (0.42-1.19) | 0.19 | 0.84  (0.50-1.40) | 0.51 | 0.57  (0.32-1.01) | 0.05 | 1  [Reference] |  |
| **Post-traumatic stress disorder symptoms** | | | | | | | | | | | | | | | | | | | | | | | | | | | | | | | | |
| Total | 1.78  (1.26-1.92) | 0.00 | 1  [Reference] |  |  | 1.37  (0.62-3.04) | 0.42 | 1.44  (0.66-3.12) | 0.34 | 1.45  (0.66-3.17) | 0.34 | 1.91  (0.78-4.67) | 0.15 | 1  [Reference] |  |  | 0.94  (0.67-1.31) | 0.72 | 1.13  (0.78-1.65) | 0.51 | 1.03  (0.69-1.53) | 0.86 | 0.91  (0.62-1.33) | 0.64 | 1.22  (0.78-1.89) | 0.37 | 1.26  (0.83-1.95) | 0.26 | 0.96  (0.58-1.58) | 0.87 | 1  [Reference] |  |
| Vaccinated  population | 1.49  (1.18-1.93) | 0.05 | 1  [Reference] |  |  | 1.19  (0.32-4.37) | 0.79 | 1.26  (0.35-4.47) | 0.71 | 1.83  (0.51-6.57) | 0.35 | 1.68  (0.41-6.92) | 0.46 | 1  [Reference] |  |  | 1.10  (0.60-2.02) | 0.73 | 1.11  (0.56-2.20) | 0.74 | 0.84  (0.39-1.78) | 0.66 | 0.79  (0.39-1.57) | 0.50 | 1.24  (0.52-2.96) | 0.62 | 0.84  (0.37-1.89) | 0.68 | 0.56  (0.20-1.56) | 0.27 | 1  [Reference] |  |
| Unvaccinated population | 0.85  (0.68-1.06) | 0.17 | 1  [Reference] |  |  | 1.65  (0.59-4.65) | 0.33 | 1.80  (0.66-4.91) | 0.24 | 1.34  (0.48-3.68) | 0.56 | 2.25  (0.68-7.46) | 0.18 | 1  [Reference] |  |  | 0.66  (0.43-1.00) | 0.51 | 1.07  (0.68-1.69) | 0.76 | 1.01  (0.63-1.62) | 0.94 | 0.93  (0.57-1.45) | 0.73 | 1.12  (0.67-1.88) | 0.65 | 1.45  (0.87-2.42) | 0.14 | 1.01  (0.56-1.80) | 0.97 | 1  [Reference] |  |
| **Insomnia symptoms** | | | | | | | | | | | | | | | | | | | | | | | | | | | | | | | |  |
| Total | 0.86  (0.72-1.02) | 0.09 | 1  [Reference] |  |  | 1.03  (0.44-2.37) | 0.93 | 0.97  (0.43-2.18) | 0.94 | 0.95  (0.42-2.15) | 0.90 | 1.10  (0.44-2.78) | 0.82 | 1  [Reference] |  |  | 0.81  (0.56-1.16) | 0.25 | 0.94  (0.63-1.40) | 0.78 | 0.88  (0.58-1.34) | 0.56 | 0.93  (0.62-1.40) | 0.74 | 0.73  (0.47-1.15) | 0.18 | 0.65  (0.42-1.01) | 0.05 | 0.71  (0.42-1.19) | 0.20 | 1  [Reference] |  |
| Vaccinated  population | 0.99  (0.73-1.35) | 0.98 | 1  [Reference] |  |  | 0.45  (0.05-3.85) | 0.46 | 0.42  (0.05-3.53) | 0.42 | 0.39  (0.04-3.35) | 0.39 | 0.44  (0.04-4.18) | 0.47 | 1  [Reference] |  |  | 1.52  (0.78-2.97) | 0.21 | 1.33  (0.62-2.83) | 0.45 | 0.83  (0.37-1.85) | 0.65 | 1.15  (0.53-2.48) | 0.70 | 0.88  (0.36-2.15) | 0.78 | 0.59  (0.25-1.38) | 0.22 | 0.70  (0.23-2.13) | 0.54 | 1  [Reference] |  |
| Unvaccinated population | 0.80  (0.64-1.00) | 0.05 | 1  [Reference] |  |  | 1.57  (0.57-4.27) | 0.37 | 1.45  (0.55-3.84) | 0.45 | 1.35  (0.50-3.60) | 0.54 | 1.69  (0.53-5.32) | 0.36 | 1  [Reference] |  |  | 1.88  (1.20-2.94) | 0.02 | 0.76  (0.48-1.22) | 0.27 | 0.84  (0.52-1.36) | 0.49 | 0.83  (0.51-1.34) | 0.45 | 0.65  (0.39-1.14) | 0.14 | 0.63  (0.38-1.04) | 0.07 | 0.63  (0.35-1.13) | 0.12 | 1  [Reference] |  |
| **Fear symptoms** | | | | | | | | |  | | | | | | | | | | | | | | | | | | | | | | | |
| Total | 0.93  (0.77-1.13) | 0.50 | 1  [Reference] |  |  | 1.76  (0.76-4.05) | 0.18 | 1.96  (0.87-4.39) | 0.10 | 1.59  (0.70-3.59) | 0.26 | 2.11  (0.82-5.44) | 0.12 | 1  [Reference] |  |  | 0.81  (0.54-1.20) | 0.30 | 0.87  (0.56-1.34) | 0.53 | 0.79  (0.50-1.25) | 0.32 | 1.02  (0.65-1.60) | 0.92 | 0.71  (0.43-1.15) | 0.16 | 0.66  (0.41-1.06) | 0.08 | 0.59  (0.34-1.02) | 0.06 | 1  [Reference] |  |
| Vaccinated  population | 0.81  (0.58-1.16) | 0.22 | 1  [Reference] |  |  | 1.40  (0.27-7.15) | 0.68 | 1.60  (0.33-7.80) | 0.55 | 1.19  (0.24-5.82) | 0.82 | 1.29  (0.23-7.20) | 0.77 | 1  [Reference] |  |  | 0.74  (0.34-1.62) | 0.46 | 0.77  (0.32-1.81) | 0.55 | 0.61  (0.24-1.56) | 0.30 | 0.89  (0.37-2.18) | 0.81 | 0.69  (0.24-1.94) | 0.48 | 0.44  (0.17-1.18) | 0.10 | 0.59  (0.17-2.09) | 0.42 | 1  [Reference] |  |
| Unvaccinated population | 1.03  (0.83-1.32) | 0.68 | 1  [Reference] |  |  | 1.90  (0.69-5.24) | 0.21 | 2.15  (0.81-5.73) | 0.12 | 1.83  (0.68-4.91) | 0.23 | 2.94  (0.87-9.92) | 0.08 | 1  [Reference] |  |  | 0.74  (0.46-1.19) | 0.21 | 0.86  (0.51-1.43) | 0.56 | 0.82  (0.48-1.36) | 0.43 | 1.02  (0.60-1.72) | 0.93 | 0.68  (0.39-1.18) | 0.17 | 0.67  (0.40-1.19) | 0.19 | 0.58  (0.31-1.05) | 0.09 | 1  [Reference] |  |

**Supplement Table S2.** Multivariate logistic regression analysis of factors associated with psychological effects among vaccinated and unvaccinated populations against COVID-19 infection (continued).

| **Psychological effects** | **Residence** | | | |  | **Family type** | | | |  | **Education level** | | | |  | **Marital status** | | | | | |  | **Having children** | | | |  | **Occupation** | | | | | | | |
| --- | --- | --- | --- | --- | --- | --- | --- | --- | --- | --- | --- | --- | --- | --- | --- | --- | --- | --- | --- | --- | --- | --- | --- | --- | --- | --- | --- | --- | --- | --- | --- | --- | --- | --- | --- |
|  | **Urban** | | **Rural** | |  | **Nuclear** | | **Joint** | |  | **College/below** | | **University/higher** | |  | **Single** | | **Married** | | **Divorced/separated/widowed** | |  | **Yes** | | **No** | |  | **Student** | | **Unemployed** | | **Employed** | | **Businessman** | |
|  | **AOR**  **(95% CI)** | ***p* value** | **AOR**  **(95% CI)** | ***p***  **value** |  | **AOR**  **(95% CI)** | ***p* value** | **AOR**  **(95% CI)** | ***p***  **value** |  | **AOR**  **(95% CI)** | ***p* value** | **AOR**  **(95% CI)** | ***p***  **value** |  | **AOR**  **(95% CI)** | ***p* value** | **AOR**  **(95% CI)** | ***p***  **value** | **AOR**  **(95% CI)** | ***p* value** |  | **AOR**  **(95% CI)** | ***p* value** | **AOR**  **(95% CI)** | ***p***  **value** |  | **AOR**  **(95% CI)** | ***p* value** | **AOR**  **(95% CI)** | ***p***  **value** | **AOR**  **(95% CI)** | ***p* value** | **AOR**  **(95% CI)** | ***p***  **value** |
| **Psychological distress symptoms** | | | | | | | | | | | | | | | | | | | |  | | | | | | |  | | | | |  |  |  |  |
| Total | 0.94  (0.80-1.11) | 0.51 | 1  [Reference] |  |  | 1.29  (1.09-1.52) | 0.00 | 1  [Reference] |  |  | 0.84  (0.71-0.99) | 0.04 | 1  [Reference] |  |  | 0.71  (0.50-1.10) | 0.14 | 0.78  (0.55-1.08) | 0.00 | 1  [Reference] |  |  | 1.00  (0.83-1.21) | 0.94 | 1  [Reference] |  |  | 1.73  (1.22-2.46) | 0.00 | 0.92  (0.63-1.33) | 0.66 | 1.07  (0.87-1.31) | 0.50 | 1.09  (0.82-1.44) | 0.54 |
| Vaccinated  population | 0.87  (0.70-1.10) | 0.26 | 1  [Reference] |  |  | 1.38  (1.09-1.77) | 0.00 | 1  [Reference] |  |  | 0.84  (0.64-1.10) | 0.20 | 1  [Reference] |  |  | 0.55  (0.25-1.20) | 0.13 | 1.44  (1.08-1.90) | 0.00 | 1  [Reference] |  |  | 1.04  (0.78-1.39) | 0.78 | 1  [Reference] |  |  | 1.23  (0.72-2.09) | 0.43 | 2.00  (1.05-3.80) | 0.04 | 1.07  (0.78-1.47) | 0.65 | 1.32  (0.84-2.07) | 0.22 |
| Unvaccinated population | 0.86  (0.71-1.04) | 0.13 | 1  [Reference] |  |  | 1.34  (1.07-1.68) | 0.01 | 1  [Reference] |  |  | 0.88  (0.70-1.08) | 0.22 | 1  [Reference] |  |  | 0.81  (0.50-1.32) | 0.41 | 0.89  (0.59-1.34) | 0.60 | 1  [Reference] |  |  | 0.97  (0.76-1.25) | 0.86 | 1  [Reference] |  |  | 2.22  (1.39-3.55) | 0.00 | 1.83  (1.10-2.62) | 0.00 | 1.06  (0.81-1.39) | 0.64 | 0.96  (0.67-1.39) | 0.85 |
| **Depression symptoms** | | | | | | | | | | | | | | | | | | | | | | | | | | | | | | | | | | | |
| Total | 1.03  (0.86-1.23) | 0.73 | 1  [Reference] |  |  | 1.71  (1.19-1.85) | 0.00 | 1  [Reference] |  |  | 1.22  (1.02-1.46) | 0.04 | 1  [Reference] |  |  | 1.27  (0.85-1.89) | 0.23 | 1.23  (0.88-1.72) | 0.22 | 1  [Reference] |  |  | 1.10  (0.90-1.35) | 0.33 | 1  [Reference] |  |  | 0.82  (0.57-1.17) | 0.28 | 0.69  (0.47-1.02) | 0.03 | 0.98  (0.78-1.22) | 0.88 | 0.79  (0.57-1.04) | 0.09 |
| Vaccinated  population | 1.09  (0.80-1.49) | 0.57 | 1  [Reference] |  |  | 1.49  (1.11-1.98) | 0.00 | 1  [Reference] |  |  | 1.39  (1.00-1.93) | 0.03 | 1  [Reference] |  |  | 1.91  (0.90-4.05) | 0.09 | 2.37  (1.72-3.24) | 0.00 | 1  [Reference] |  |  | 1.17  (1.01-1.69) | 0.00 | 1  [Reference] |  |  | 0.74  (0.40-1.36) | 0.34 | 0.83  (0.42-1.61) | 0.58 | 1.01  (0.67-1.53) | 0.93 | 0.99  (0.55-1.77) | 0.98 |
| Unvaccinated population | 0.94  (0.75-1.18) | 0.62 | 1  [Reference] |  |  | 0.87  (0.69-1.09) | 0.24 | 1  [Reference] |  |  | 1.18  (0.95-1.49) | 0.12 | 1  [Reference] |  |  | 0.96  (0.59-1.55) | 0.88 | 0.83  (0.55-1.24) | 0.37 | 1  [Reference] |  |  | 1.66  (1.21-2.18) | 0.00 | 1  [Reference] |  |  | 0.93  (0.59-1.48) | 0.78 | 1.74  (1.37-2.19) | 0.03 | 1.02  (0.78-1.34) | 0.85 | 0.71  (0.49-1.03) | 0.07 |
| **Anxiety symptoms** | | | | | | | | | | | | | | | | | | | | | | | | | | | | | | | | | | | |
| Total | 1.15  (0.97-1.38) | 0.10 | 1  [Reference] |  |  | 1.72  (1.34-1.86) | 0.00 | 1  [Reference] |  |  | 0.80  (0.67-0.95) | 0.01 | 1  [Reference] |  |  | 0.85  (0.57-1.01) | 0.00 | 1.04  (0.74-1.47) | 0.79 | 1  [Reference] |  |  | 1.20  (0.98-1.46) | 0.00 | 1  [Reference] |  |  | 0.73  (0.51-1.04) | 0.08 | 1.04  (0.69-1.57) | 0.00 | 0.84  (0.68-1.05) | 0.13 | 1.05  (0.77-1.42) | 0.74 |
| Vaccinated  population | 1.15  (0.84-1.56) | 0.36 | 1  [Reference] |  |  | 1.69  (1.21-1.98) | 0.00 | 1  [Reference] |  |  | 0.73  (0.53-0.99) | 0.02 | 1  [Reference] |  |  | 0.72  (0.32-1.62) | 0.43 | 1.57  (1.14-2.16) | 0.00 | 1  [Reference] |  |  | 1.14  (0.80-1.63) | 0.44 | 1  [Reference] |  |  | 1.17  (0.65-2.10) | 0.58 | 1.14  (0.81-1.60) | 0.86 | 1.18  (0.85-1.65) | 0.19 | 1.47  (1.05-2.06) | 0.92 |
| Unvaccinated population | 1.14  (0.91-1.44) | 0.23 | 1  [Reference] |  |  | 0.68  (0.54-0.86) | 0.00 | 1  [Reference] |  |  | 0.84  (0.68-1.05) | 0.14 | 1  [Reference] |  |  | 0.74  (0.45-1.21) | 0.23 | 0.88  (0.58-1.00) | 0.00 | 1  [Reference] |  |  | 1.49  (1.12-1.87) | 0.03 | 1  [Reference] |  |  | 0.77  (0.49-1.23) | 0.28 | 1.70  (1.12-2.11) | 0.00 | 0.96  (0.73-1.26) | 0.77 | 1.10  (0.75-1.60) | 0.61 |
| **Stress symptoms** | | | | | | | | | | | | | | | | | | | | | | | | | | | | | | | | | | | |
| Total | 0.95  (0.79-1.15) | 0.65 | 1  [Reference] |  |  | 0.99  (0.82-1.19) | 0.95 | 1  [Reference] |  |  | 1.01  (0.84-1.22) | 0.85 | 1  [Reference] |  |  | 1.59  (1.05-2.40) | 0.04 | 1.40  (0.99-1.98) | 0.05 | 1  [Reference] |  |  | 1.20  (0.98-1.48) | 0.03 | 1  [Reference] |  |  | 0.91  (0.62-1.33) | 0.63 | 1.17  (0.75-1.81) | 0.47 | 0.89  (0.70-1.12) | 0.33 | 0.82  (0.60-1.12) | 0.22 |
| Vaccinated  population | 0.89  (0.65-1.23) | 0.49 | 1  [Reference] |  |  | 0.87  (0.64-1.19) | 0.40 | 1  [Reference] |  |  | 0.76  (0.55-1.05) | 0.10 | 1  [Reference] |  |  | 1.82  (0.81-4.07) | 0.14 | 1.53  (0.77-3.04) | 0.21 | 1  [Reference] |  |  | 1.21  (0.85-1.73) | 0.27 | 1  [Reference] |  |  | 1.18  (0.59-2.36) | 0.63 | 1.50  (1.22-3.45) | 0.00 | 0.69  (0.43-1.00) | 0.05 | 0.63  (0.36-1.11) | 0.11 |
| Unvaccinated population | 0.98  (0.77-1.24) | 0.86 | 1  [Reference] |  |  | 1.04  (0.82-1.32) | 0.70 | 1  [Reference] |  |  | 1.16  (0.92-1.46) | 0.20 | 1  [Reference] |  |  | 1.46  (0.89-2.40) | 0.13 | 1.29  (0.85-1.95) | 0.22 | 1  [Reference] |  |  | 1.43  (1.07-1.83) | 0.02 | 1  [Reference] |  |  | 0.81  (0.50-1.30) | 0.39 | 1.46  (1.08-1.92) | 0.00 | 1.07  (0.78-1.39) | 0.75 | 0.95  (0.64-1.40) | 0.81 |
| **Post-traumatic stress disorder symptoms** | | | | | | | | | | | | | | | | | | | | | | | | | | | | | | | | | | | |
| Total | 1.04  (0.88-1.24) | 0.59 | 1  [Reference] |  |  | 0.96  (0.81-1.14) | 0.67 | 1  [Reference] |  |  | 0.95  (0.80-1.12) | 0.56 | 1  [Reference] |  |  | 0.87  (0.58-1.29) | 0.50 | 1.00  (0.71-1.41) | 0.96 | 1  [Reference] |  |  | 1.24  (1.02-1.51) | 0.04 | 1  [Reference] |  |  | 1.12  (0.79-1.59) | 0.49 | 1.26  (0.85-1.87) | 0.24 | 0.99  (0.81-1.23) | 0.99 | 1.09  (0.81-1.46) | 0.53 |
| Vaccinated  population | 1.09  (0.82-1.43) | 0.54 | 1  [Reference] |  |  | 0.88  (0.66-1.16) | 0.36 | 1  [Reference] |  |  | 0.97  (0.73-1.29) | 0.87 | 1  [Reference] |  |  | 0.91  (0.44-1.88) | 0.81 | 1.26  (1.06-1.52) | 0.00 | 1  [Reference] |  |  | 1.03  (0.75-1.42) | 0.81 | 1  [Reference] |  |  | 0.98  (0.57-1.68) | 0.94 | 1.08  (0.58-2.02) | 0.79 | 1.06  (0.75-1.50) | 0.73 | 1.12  (0.68-1.85) | 0.64 |
| Unvaccinated population | 0.96  (0.77-1.21) | 0.78 | 1  [Reference] |  |  | 1.06  (0.84-1.33) | 0.61 | 1  [Reference] |  |  | 0.95  (0.76-1.19) | 0.68 | 1  [Reference] |  |  | 0.81  (0.49-1.32) | 0.40 | 0.86  (0.56-1.30) | 0.48 | 1  [Reference] |  |  | 1.32  (1.05-1.71) | 0.05 | 1  [Reference] |  |  | 1.37  (0.86-2.17) | 0.18 | 1.61  (0.94-2.75) | 0.09 | 1.02  (0.78-1.33) | 0.87 | 1.13  (0.78-1.64) | 0.50 |
| **Insomnia symptoms** | | | | | | | | | | | | | | | | | | | | | | | | | | | | | | | | | | | |
| Total | 1.14  (0.95-1.36) | 0.14 | 1  [Reference] |  |  | 1.01  (0.84-1.22) | 0.84 | 1  [Reference] |  |  | 1.09  (0.91-1.30) | 0.35 | 1  [Reference] |  |  | 2.40  (1.62-3.57) | 0.00 | 2.15  (1.55-3.00) | 0.00 | 1  [Reference] |  |  | 1.09  (0.89-1.34) | 0.36 | 1  [Reference] |  |  | 0.93  (0.63-1.35) | 0.70 | 0.64  (0.43-0.95) | 0.04 | 0.86  (0.68-1.09) | 0.19 | 0.63  (0.47-0.85) | 0.00 |
| Vaccinated  population | 1.23  (0.90-1.69) | 0.18 | 1  [Reference] |  |  | 0.99  (0.72-1.36) | 0.96 | 1  [Reference] |  |  | 0.94  (0.67-1.30) | 0.71 | 1  [Reference] |  |  | 1.85  (0.85-4.03) | 0.11 | 1.72  (1.27-2.29) | 0.00 | 1  [Reference] |  |  | 1.24  (0.86-1.79) | 0.23 | 1  [Reference] |  |  | 1.41  (0.70-2.84) | 0.32 | 1.28  (1.01-1.54) | 0.03 | 0.97  (0.64-1.45) | 0.88 | 0.91  (0.52-1.60) | 0.76 |
| Unvaccinated population | 1.05  (0.84-1.31) | 0.65 | 1  [Reference] |  |  | 1.07  (0.85-1.34) | 0.56 | 1  [Reference] |  |  | 1.16  (0.93-1.44) | 0.17 | 1  [Reference] |  |  | 2.32  (1.44-3.73) | 0.00 | 2.08  (1.39-3.10) | 0.00 | 1  [Reference] |  |  | 1.02  (0.79-1.31) | 0.85 | 1  [Reference] |  |  | 0.79  (0.50-1.26) | 0.33 | 1.87  (1.20-2.46) | 0.02 | 0.87  (0.66-1.14) | 0.32 | 0.54  (0.37-0.78) | 0.00 |
| **Fear symptoms** | | | | | | | | | | | | | | | | | | | | | | | | | | | | | | | | | | | |
| Total | 0.90  (0.74-1.09) | 0.30 | 1  [Reference] |  |  | 0.94  (0.77-1.14) | 0.00 | 1  [Reference] |  |  | 0.93  (0.77-1.13) | 0.51 | 1  [Reference] |  |  | 1.12  (0.72-1.74) | 0.59 | 1.03  (0.71-1.50) | 0.83 | 1  [Reference] |  |  | 1.02  (0.82-1.27) | 0.85 | 1  [Reference] |  |  | 0.97  (0.66-1.45) | 0.91 | 1.14  (0.73-1.79) | 0.54 | 0.99  (0.78-1.26) | 0.96 | 0.99  (0.71-1.38) | 0.98 |
| Vaccinated  population | 0.89  (0.64-1.25) | 0.52 | 1  [Reference] |  |  | 1.43  (1.11-1.82) | 0.00 | 1  [Reference] |  |  | 0.88  (0.62-1.24) | 0.47 | 1  [Reference] |  |  | 1.28  (0.52-3.19) | 0.58 | 1.03  (0.47-2.27) | 0.93 | 1  [Reference] |  |  | 1.04  (0.71-1.53) | 0.81 | 1  [Reference] |  |  | 0.75  (0.39-1.44) | 0.39 | 1.62  (0.71-3.68) | 0.24 | 1.05  (0.68-1.62) | 0.80 | 1.15  (0.62-2.11) | 0.65 |
| Unvaccinated population | 0.88  (0.69-1.13) | 0.33 | 1  [Reference] |  |  | 1.04  (0.82-1.33) | 0.71 | 1  [Reference] |  |  | 0.95  (0.75-1.21) | 0.70 | 1  [Reference] |  |  | 0.99  (0.59-1.67) | 0.99 | 0.94  (0.63-1.51) | 0.94 | 1  [Reference] |  |  | 0.99  (0.75-1.30) | 0.95 | 1  [Reference] |  |  | 1.20  (0.72-1.99) | 0.46 | 0.97  (0.56-1.69) | 0.93 | 1.03  (0.75-1.35) | 0.93 | 0.95  (0.63-1.39) | 0.75 |

| **Psychological effects** |  | | | |  | **Socioeconomic status** | | | | | |  | **Physical exercise** | | | | **Smoking habit** | | | | |  | **Alcohol use** | | | |  | **Chronic diseases** | | | |  | **Social media use** | | | |
| --- | --- | --- | --- | --- | --- | --- | --- | --- | --- | --- | --- | --- | --- | --- | --- | --- | --- | --- | --- | --- | --- | --- | --- | --- | --- | --- | --- | --- | --- | --- | --- | --- | --- | --- | --- | --- |
|  | **Housewife** | | **Other** | |  | **Lower** | | **Middle** | | **Upper** | |  | **Yes** | | **No** | |  | **Yes** | | **No** | |  | **Yes** | | **No** | |  | **Yes** | | **No** | |  | **Yes** | | **No** | |
|  | **AOR**  **(95% CI)** | ***p* value** | **AOR**  **(95% CI)** | ***p***  **value** |  | **AOR**  **(95% CI)** | ***p* value** | **AOR**  **(95% CI)** | ***p***  **value** | **AOR**  **(95% CI)** | ***p* value** |  | **AOR**  **(95% CI)** | ***p* value** | **AOR**  **(95% CI)** | ***p***  **value** |  | **AOR**  **(95% CI)** | ***p* value** | **AOR**  **(95% CI)** | ***p***  **value** |  | **AOR**  **(95% CI)** | ***p* value** | **AOR**  **(95% CI)** | ***p***  **value** |  | **AOR**  **(95% CI)** | ***p* value** | **AOR**  **(95% CI)** | ***p***  **value** |  | **AOR**  **(95% CI)** | ***p* value** | **AOR**  **(95% CI)** | ***p***  **value** |
| **Psychological distress symptoms** | | | | | | | | | | | | | | | | | | | | | | | |  | | | | | | | | | | | | |
| Total | 2.17  (1.33-3.56) | 0.00 | 1  [Reference] |  |  | 1.20  (0.88-1.65) | 0.24 | 0.78  (0.65-0.93) | 0.00 | 1  [Reference] |  |  | 0.92  (0.77-1.10) | 0.38 | 1  [Reference] |  |  | 1.35  (1.12-1.62) | 0.00 | 1  [Reference] |  |  | 1.52  (1.36-1.74) | 0.00 | 1  [Reference] |  |  | 1.05  (0.84-1.30) | 0.64 | 1  [Reference] |  |  | 1.06  (0.90-1.26) | 0.44 | 1  [Reference] |  |
| Vaccinated  population | 2.74  (1.27-5.92) | 0.01 | 1  [Reference] |  |  | 1.19  (0.71-1.98) | 0.49 | 0.85  (0.65-1.12) | 0.27 | 1  [Reference] |  |  | 1.01  (0.76-1.33) | 0.94 | 1  [Reference] |  |  | 1.67  (1.25-2.23) | 0.00 | 1  [Reference] |  |  | 0.72  (0.43-1.22) | 0.23 | 1  [Reference] |  |  | 0.93  (0.72-1.22) | 0.63 | 1  [Reference] |  |  | 1.75  (1.18-2.01) | 0.00 | 1  [Reference] |  |
| Unvaccinated population | 1.74  (0.89-3.42) | 0.10 | 1  [Reference] |  |  | 1.48  (1.01-2.16) | 0.18 | 0.74  (0.58-0.94) | 0.01 | 1  [Reference] |  |  | 0.85  (0.67-1.08) | 0.20 | 1  [Reference] |  |  | 1.12  (0.87-1.44) | 0.37 | 1  [Reference] |  |  | 2.01  (1.23-2.78) | 0.00 | 1  [Reference] |  |  | 1.32  (0.90-1.93) | 0.15 | 1  [Reference] |  |  | 1.04  (0.83-1.31) | 0.68 | 1  [Reference] |  |
| **Depression symptoms** | | | | | | | | | | | | | | | | | | | | | | | | | | | | |  | | | | | | | |
| Total | 0.93  (0.56-1.54) | 0.78 | 1  [Reference] |  |  | 1.10  (0.78-1.54) | 0.57 | 0.85  (0.70-1.03) | 0.10 | 1  [Reference] |  |  | 1.18  (0.98-1.43) | 0.07 | 1  [Reference] |  |  | 1.03  (0.84-1.25) | 0.76 | 1  [Reference] |  |  | 0.84  (0.59-1.22) | 0.37 | 1  [Reference] |  |  | 0.97  (0.77-1.22) | 0.81 | 1  [Reference] |  |  | 1.00  (0.84-1.20) | 0.95 | 1  [Reference] |  |
| Vaccinated  population | 0.82  (0.37-1.82) | 0.63 | 1  [Reference] |  |  | 1.24  (0.68-2.26) | 0.47 | 0.76  (0.54-1.07) | 0.11 | 1  [Reference] |  |  | 0.96  (0.68-1.35) | 0.83 | 1  [Reference] |  |  | 1.10  (0.78-1.53) | 0.57 | 1  [Reference] |  |  | 0.94  (0.46-1.51) | 0.57 | 1  [Reference] |  |  | 1.90  (1.26-2.23) | 0.02 | 1  [Reference] |  |  | 1.48  (1.21-1.83) | 0.00 | 1  [Reference] |  |
| Unvaccinated population | 1.07  (0.54-2.11) | 0.83 | 1  [Reference] |  |  | 1.05  (0.69-1.58) | 0.81 | 0.90  (0.71-1.14) | 0.41 | 1  [Reference] |  |  | 1.27  (1.00-1.61) | 0.04 | 1  [Reference] |  |  | 0.99  (0.77-1.28) | 0.98 | 1  [Reference] |  |  | 1.96  (1.17-2.41) | 0.03 | 1  [Reference] |  |  | 1.75  (1.16-2.01) | 0.02 | 1  [Reference] |  |  | 1.24  (0.98-1.55) | 0.00 | 1  [Reference] |  |
| **Anxiety symptoms** | | | | | | | | | | | | | | | | | | | | | | | |  |  |  |  |  |  |  |  |  |  |  |  |  |
| Total | 1.51  (0.87-1.62) | 0.14 | 1  [Reference] |  |  | 1.21  (0.87-1.67) | 0.24 | 1.29  (1.06-1.56) | 0.00 | 1  [Reference] |  |  | 1.13  (0.94-1.37) | 0.17 | 1  [Reference] |  |  | 1.13  (0.93-1.38) | 0.19 | 1  [Reference] |  |  | 1.32  (0.90-1.94) | 0.15 | 1  [Reference] |  |  | 1.33  (1.05-1.69) | 0.01 | 1  [Reference] |  |  | 1.07  (0.90-1.28) | 0.00 | 1  [Reference] |  |
| Vaccinated  population | 1.80  (0.68-4.75) | 0.23 | 1  [Reference] |  |  | 1.17  (0.65-2.10) | 0.58 | 1.14  (0.81-1.60) | 0.43 | 1  [Reference] |  |  | 1.18  (0.85-1.75) | 0.31 | 1  [Reference] |  |  | 1.47  (1.05-2.06) | 0.05 | 1  [Reference] |  |  | 0.99  (0.54-1.81) | 0.98 | 1  [Reference] |  |  | 1.13  (0.83-1.55) | 0.42 | 1  [Reference] |  |  | 1.57  (1.16-1.87) | 0.00 | 1  [Reference] |  |
| Unvaccinated population | 1.39  (0.68-2.82) | 0.35 | 1  [Reference] |  |  | 1.19  (0.79-1.80) | 0.38 | 1.39  (1.09-1.78) | 0.00 | 1  [Reference] |  |  | 1.07  (0.84-1.36) | 0.55 | 1  [Reference] |  |  | 0.97  (0.75-1.25) | 0.82 | 1  [Reference] |  |  | 2.28  (1.27-3.03) | 0.02 | 1  [Reference] |  |  | 1.51  (1.07-1.82) | 0.00 | 1  [Reference] |  |  | 1.08  (0.86-1.36) | 0.46 | 1  [Reference] |  |
| **Stress symptoms** | | | | | | | | | | | | | | | | | | | | | | | | | | | | | | | | | | | | |
| Total | 0.55  (0.34-0.90) | 0.01 | 1  [Reference] |  |  | 1.08  (0.75-1.54) | 0.66 | 0.89  (0.73-1.09) | 0.28 | 1  [Reference] |  |  | 1.27  (1.04-1.56) | 0.01 | 1  [Reference] |  |  | 0.93  (0.76-1.14) | 0.51 | 1  [Reference] |  |  | 1.17  (0.78-1.75) | 0.42 | 1  [Reference] |  |  | 0.90  (0.71-1.15) | 0.43 | 1  [Reference] |  |  | 1.04  (0.87-1.25) | 0.63 | 1  [Reference] |  |
| Vaccinated  population | 0.49  (0.23-1.03) | 0.06 | 1  [Reference] |  |  | 1.17  (0.60-2.26) | 0.63 | 0.70  (0.50-0.97) | 0.03 | 1  [Reference] |  |  | 1.36  (0.95-1.94) | 0.08 | 1  [Reference] |  |  | 1.13  (0.80-1.59) | 0.48 | 1  [Reference] |  |  | 1.34  (1.14-2.72) | 0.03 | 1  [Reference] |  |  | 0.99  (0.72-1.37) | 0.97 | 1  [Reference] |  |  | 0.96  (0.70-1.30) | 0.80 | 1  [Reference] |  |
| Unvaccinated population | 0.55  (0.28-1.08) | 0.08 | 1  [Reference] |  |  | 0.99  (0.64-1.53) | 0.97 | 1.05  (0.79-1.31) | 0.88 | 1  [Reference] |  |  | 1.22  (0.95-1.59) | 0.11 | 1  [Reference] |  |  | 0.84  (0.64-1.09) | 0.19 | 1  [Reference] |  |  | 1.83  (1.04-2.66) | 0.04 | 1  [Reference] |  |  | 0.84  (0.57-1.25) | 0.38 | 1  [Reference] |  |  | 1.16  (0.91-1.47) | 0.22 | 1  [Reference] |  |
| **Post-traumatic stress disorder symptoms** | | | | | | | | | | |  | | | | | | | | | | | | | | | | | | | | | | | | | |
| Total | 1.46  (0.87-2.46) | 0.15 | 1  [Reference] |  |  | 0.83  (0.61-1.13) | 0.24 | 0.97  (0.81-1.17) | 0.80 | 1  [Reference] |  |  | 1.22  (1.02-1.46) | 0.04 | 1  [Reference] |  |  | 0.95  (0.77-1.13) | 0.50 | 1  [Reference] |  |  | 1.08  (0.75-1.55) | 0.66 | 1  [Reference] |  |  | 1.55  (0.92-1.44) | 0.19 | 1  [Reference] |  |  | 0.95  (0.80-1.13) | 0.59 | 1  [Reference] |  |
| Vaccinated  population | 1.59  (0.72-3.50) | 0.24 | 1  [Reference] |  |  | 0.68  (0.41-1.13) | 0.14 | 0.95  (0.70-1.29) | 0.77 | 1  [Reference] |  |  | 1.06  (0.79-1.43) | 0.67 | 1  [Reference] |  |  | 0.98  (0.73-1.32) | 0.93 | 1  [Reference] |  |  | 0.83  (0.48-1.42) | 0.50 | 1  [Reference] |  |  | 1.02  (0.77-1.36) | 0.84 | 1  [Reference] |  |  | 0.85  (0.65-1.12) | 0.25 | 1  [Reference] |  |
| Unvaccinated population | 1.46  (0.71-3.00) | 0.29 | 1  [Reference] |  |  | 0.90  (0.60-1.34) | 0.61 | 0.98  (0.77-1.25) | 0.89 | 1  [Reference] |  |  | 1.29  (1.01-1.63) | 0.02 | 1  [Reference] |  |  | 0.90  (0.70-1.18) | 0.44 | 1  [Reference] |  |  | 1.41  (0.85-2.36) | 0.18 | 1  [Reference] |  |  | 1.98  (1.12-2.41) | 0.05 | 1  [Reference] |  |  | 1.04  (0.83-1.31) | 0.68 | 1  [Reference] |  |
| **Insomnia symptoms** | | | | | | | | | | | | | | | | | | | | | | | | | | | | | | | | | | | | |
| Total | 0.71  (0.43-1.18) | 0.19 | 1  [Reference] |  |  | 1.04  (0.74-1.47) | 0.81 | 1.00  (0.82-1.21) | 0.95 | 1  [Reference] |  |  | 1.10  (0.91-1.33) | 0.30 | 1  [Reference] |  |  | 0.84  (0.69-1.02) | 0.08 | 1  [Reference] |  |  | 1.19  (0.81-1.75) | 0.35 | 1  [Reference] |  |  | 1.06  (0.83-1.35) | 0.62 | 1  [Reference] |  |  | 0.89  (0.74-1.06) | 0.21 | 1  [Reference] |  |
| Vaccinated  population | 2.09  (0.80-5.46) | 0.12 | 1  [Reference] |  |  | 0.92  (0.52-1.65) | 0.80 | 1.21  (0.84-1.72) | 0.29 | 1  [Reference] |  |  | 1.17  (0.82-1.66) | 0.37 | 1  [Reference] |  |  | 0.82  (0.59-1.16) | 0.27 | 1  [Reference] |  |  | 0.94  (0.51-1.73) | 0.84 | 1  [Reference] |  |  | 1.19  (0.85-1.65) | 0.29 | 1  [Reference] |  |  | 1.68  (1.02-1.97) | 0.01 | 1  [Reference] |  |
| Unvaccinated population | 0.41  (0.21-0.80) | 0.00 | 1  [Reference] |  |  | 1.17  (0.77-1.77) | 0.44 | 0.94  (0.74-1.19) | 0.62 | 1  [Reference] |  |  | 1.06  (0.84-1.34) | 0.59 | 1  [Reference] |  |  | 0.85  (0.66-1.09) | 0.22 | 1  [Reference] |  |  | 1.46  (0.89-2.40) | 0.12 | 1  [Reference] |  |  | 0.89  (0.61-1.30) | 0.56 | 1  [Reference] |  |  | 1.06  (0.85-1.33) | 0.56 | 1  [Reference] |  |
| **Fear symptoms** | | | | | | | | | | | | | | | | | | | | | | | | | | | | | | | | | | | |  |
| Total | 0.78  (0.46-1.31) | 0.35 | 1  [Reference] |  |  | 1.42  (0.97-2.10) | 0.06 | 0.90  (0.73-1.10) | 0.32 | 1  [Reference] |  |  | 0.93  (0.76-1.14) | 0.52 | 1  [Reference] |  |  | 0.81  (0.66-1.00) | 0.05 | 1  [Reference] |  |  | 0.82  (0.56-1.20) | 0.31 | 1  [Reference] |  |  | 1.11  (0.86-1.44) | 0.41 | 1  [Reference] |  |  | 0.99  (0.82-1.20) | 0.93 | 1  [Reference] |  |
| Vaccinated  population | 0.59  (0.27-1.28) | 0.18 | 1  [Reference] |  |  | 1.39  (0.72-2.69) | 0.32 | 1.09  (0.76-1.57) | 0.62 | 1  [Reference] |  |  | 0.72  (0.51-1.03) | 0.07 | 1  [Reference] |  |  | 0.80  (0.56-1.13) | 0.21 | 1  [Reference] |  |  | 0.93  (0.49-1.75) | 0.82 | 1  [Reference] |  |  | 1.06  (0.75-1.50) | 0.70 | 1  [Reference] |  |  | 0.75  (0.54-1.04) | 0.09 | 1  [Reference] |  |
| Unvaccinated population | 1.09  (0.53-2.26) | 0.80 | 1  [Reference] |  |  | 1.45  (0.90-2.34) | 0.12 | 0.85  (0.63-1.04) | 0.11 | 1  [Reference] |  |  | 1.01  (0.79-1.31) | 0.88 | 1  [Reference] |  |  | 0.84  (0.64-1.09) | 0.20 | 1  [Reference] |  |  | 0.79  (0.48-1.29) | 0.35 | 1  [Reference] |  |  | 1.15  (0.76-1.75) | 0.48 | 1  [Reference] |  |  | 1.17  (0.92-1.49) | 0.19 | 1  [Reference] |  |

**Supplement Table S2.** Multivariate logistic regression analysis of factors associated with psychological effects among vaccinated and unvaccinated populations against COVID-19 infection (continued).

**Supplement Table S2.** Multivariate logistic regression analysis of factors associated with psychological effects among vaccinated and unvaccinated populations against COVID-19 infection (continued).

| **Psychological effects** | **Have you been infected with COVID-19?** | | | |  | **Have any of your family members, friends, or colleagues been infected with COVID-19?** | | | |  | **Have any of your family members, friends, or colleagues died of COVID-19?** | | | |  | **Have you been vaccinated against the COVID-19 infection?** | | | |  | **Social support** | | | | | |
| --- | --- | --- | --- | --- | --- | --- | --- | --- | --- | --- | --- | --- | --- | --- | --- | --- | --- | --- | --- | --- | --- | --- | --- | --- | --- | --- |
|  | **Yes** | | **No** | |  | **Yes** | | **No** | |  | **Yes** | | **No** | |  | **Yes** | | **No** | |  | **Poor** | | **Moderate** | | **Strong** | |
|  | **AOR**  **(95% CI)** | ***p* value** | **AOR**  **(95% CI)** | ***p***  **value** |  | **AOR**  **(95% CI)** | ***p* value** | **AOR**  **(95% CI)** | ***p***  **value** |  | **AOR**  **(95% CI)** | ***p* value** | **AOR**  **(95% CI)** | ***p***  **value** |  | **AOR**  **(95% CI)** | ***p* value** | **AOR**  **(95% CI)** | ***p***  **value** |  | **AOR**  **(95% CI)** | ***p* value** | **AOR**  **(95% CI)** | ***p***  **value** | **AOR**  **(95% CI)** | ***p* value** |
| **Psychological distress symptoms** | | | | | | | | | | | | | | | | | | | | | | | | | | |
| Total | 0.89  (0.75-1.07) | 0.22 | 1  [Reference] |  |  | 1.08  (0.92-1.27) | 0.33 | 1  [Reference] |  |  | 1.87  (1.56-2.24) | 0.00 | 1  [Reference] |  |  | 1.72  (1.44-2.05) | 0.00 | 1  [Reference] |  |  | 0.86  (0.68-1.08) | 0.20 | 0.83  (0.67-1.03) | 0.10 | 1  [Reference] |  |
| Vaccinated  population | 1.00  (0.76-1.31) | 0.99 | 1  [Reference] |  |  | 2.01  (1.33-2.86) | 0.00 | 1  [Reference] |  |  | 1.35  (1.02-1.79) | 0.02 | 1  [Reference] |  |  | - | - | 1  [Reference] |  |  | 0.64  (0.41-1.00) | 0.05 | 0.62  (0.41-0.94) | 0.45 | 1  [Reference] |  |
| Unvaccinated population | 1.66  (1.23-2.02) | 0.03 | 1  [Reference] |  |  | 1.03  (0.82-1.29) | 0.74 | 1  [Reference] |  |  | 2.48  (1.93-3.23) | 0.00 | 1  [Reference] |  |  | - | - | 1  [Reference] |  |  | 1.00  (0.76-1.32) | 0.98 | 1.00  (0.77-1.30) | 0.97 | 1  [Reference] |  |
| **Depression symptoms** | | | | | | | | | | | | | | | | | | | | | | | | | | |
| Total | 1.04  (0.86-1.25) | 0.04 | 1  [Reference] |  |  | 0.88  (0.74-1.05) | 0.16 | 1  [Reference] |  |  | 0.88  (0.73-1.07) | 0.21 | 1  [Reference] |  |  | 2.16  (1.78-2.62) | 0.00 | 1  [Reference] |  |  | 1.41  (1.12-1.78) | 0.00 | 1.27  (1.03-1.58) | 0.03 | 1  [Reference] |  |
| Vaccinated  population | 0.86  (0.62-1.18) | 0.36 | 1  [Reference] |  |  | 0.76  (0.47-0.94) | 0.04 | 1  [Reference] |  |  | 0.73  (0.53-1.00) | 0.51 | 1  [Reference] |  |  | - | - | 1  [Reference] |  |  | 0.90  (0.57-1.43) | 0.67 | 1.23  (0.80-1.90) | 0.33 | 1  [Reference] |  |
| Unvaccinated population | 1.79  (1.07-2.01) | 0.03 | 1  [Reference] |  |  | 0.98  (0.78-1.22) | 0.86 | 1  [Reference] |  |  | 1.03  (0.80-1.33) | 0.76 | 1  [Reference] |  |  | - | - | 1  [Reference] |  |  | 1.88  (1.35-2.41) | 0.00 | 1.16  (0.89-1.50) | 0.25 | 1  [Reference] |  |
| **Anxiety symptoms** | | | | | | | | | | | | | | | | | | | | | | | | | | |
| Total | 1.12  (0.93-1.37) | 0.22 | 1  [Reference] |  |  | 0.76  (0.64-0.90) | 0.00 | 1  [Reference] |  |  | 0.53  (0.44-0.64) | 0.00 | 1  [Reference] |  |  | 2.62  (2.17-3.16) | 0.00 | 1  [Reference] |  |  | 1.56  (0.44-1.71) | 0.00 | 0.66  (0.52-0.83) | 0.00 | 1  [Reference] |  |
| Vaccinated  population | 1.38  (1.00-1.90) | 0.02 | 1  [Reference] |  |  | 0.54  (0.21-0.81) | 0.00 | 1  [Reference] |  |  | 1.41  (1.11-1.87) | 0.04 | 1  [Reference] |  |  | - | - | 1  [Reference] |  |  | 1.06  (0.41-1.26) | 0.08 | 1.11  (0.71-1.74) | 0.62 | 1  [Reference] |  |
| Unvaccinated population | 1.61  (1.22-1.98) | 0.02 | 1  [Reference] |  |  | 1.00  (0.80-1.26) | 0.96 | 1  [Reference] |  |  | 0.87  (0.67-1.12) | 0.29 | 1  [Reference] |  |  | - | - | 1  [Reference] |  |  | 1.48  (1.01-1.81) | 0.02 | 0.47  (0.35-0.62) | 0.00 | 1  [Reference] |  |
| **Stress symptoms** | | | | | | | | | | | | | | | | | | | | | | | | | | |
| Total | 1.13  (0.92-1.38) | 0.22 | 1  [Reference] |  |  | 0.99  (0.82-1.19) | 0.93 | 1  [Reference] |  |  | 1.15  (0.94-1.42) | 0.15 | 1  [Reference] |  |  | 1.68  (1.37-2.05) | 0.00 | 1  [Reference] |  |  | 1.06  (0.83-1.36) | 0.61 | 1.01  (0.80-1.27) | 0.90 | 1  [Reference] |  |
| Vaccinated  population | 1.07  (0.76-1.48) | 0.68 | 1  [Reference] |  |  | 0.87  (0.64-1.18) | 0.38 | 1  [Reference] |  |  | 1.04  (0.74-1.46) | 0.81 | 1  [Reference] |  |  | - | - | 1  [Reference] |  |  | 2.45  (1.53-3.93) | 0.09 | 2.19  (1.44-3.32) | 0.16 | 1  [Reference] |  |
| Unvaccinated population | 1.14  (0.88-1.47) | 0.30 | 1  [Reference] |  |  | 1.08  (0.85-1.37) | 0.50 | 1  [Reference] |  |  | 1.22  (0.94-1.60) | 0.13 | 1  [Reference] |  |  | - | - | 1  [Reference] |  |  | 0.80  (0.60-1.08) | 0.15 | 0.76  (0.58-1.01) | 0.06 | 1  [Reference] |  |
| **Post-traumatic stress disorder symptoms** | | | | | | | | | | | | | | | | | | | | | | | | | | |
| Total | 0.85  (0.71-1.02) | 0.09 | 1  [Reference] |  |  | 0.63  (0.54-0.75) | 0.00 | 1  [Reference] |  |  | 0.86  (0.72-1.03) | 0.11 | 1  [Reference] |  |  | 1.60  (1.33-1.92) | 0.00 | 1  [Reference] |  |  | 0.62  (0.49-0.79) | 0.00 | 0.69  (0.55-0.86) | 0.00 | 1  [Reference] |  |
| Vaccinated  population | 0.84  (0.63-1.11) | 0.22 | 1  [Reference] |  |  | 1.59  (1.10-1.86) | 0.00 | 1  [Reference] |  |  | 1.76  (1.24-2.19) | 0.02 | 1  [Reference] |  |  | - | - | 1  [Reference] |  |  | 1.39  (1.08-1.62) | 0.00 | 1.52  (1.33-1.81) | 0.00 | 1  [Reference] |  |
| Unvaccinated population | 0.85  (0.66-1.06) | 0.15 | 1  [Reference] |  |  | 1.73  (1.34-1.91) | 0.00 | 1  [Reference] |  |  | 1.05  (0.82-1.35) | 0.68 | 1  [Reference] |  |  | - | - | 1  [Reference] |  |  | 1.71  (1.20-2.09) | 0.02 | 1.96  (1.53-1.90) | 0.00 | 1  [Reference] |  |
| **Insomnia symptoms** | | | | | | | | | | | | | | | | | | | | | | | | | | |
| Total | 0.99  (0.82-1.20) | 0.96 | 1  [Reference] |  |  | 0.85  (0.71-1.02) | 0.08 | 1  [Reference] |  |  | 0.74  (0.61-0.90) | 0.00 | 1  [Reference] |  |  | 2.94  (2.41-3.59) | 0.00 | 1  [Reference] |  |  | 1.16  (0.92-1.47) | 0.20 | 1.13  (0.90-1.40) | 0.27 | 1  [Reference] |  |
| Vaccinated  population | 1.05  (0.76-1.46) | 0.73 | 1  [Reference] |  |  | 0.97  (0.71-1.32) | 0.88 | 1  [Reference] |  |  | 0.87  (0.62-1.22) | 0.43 | 1  [Reference] |  |  | - | - | 1  [Reference] |  |  | 1.25  (0.79-1.97) | 0.33 | 1.61  (1.05-2.47) | 0.03 | 1  [Reference] |  |
| Unvaccinated population | 0.97  (0.77-1.24) | 0.86 | 1  [Reference] |  |  | 0.85  (0.68-1.07) | 0.17 | 1  [Reference] |  |  | 0.75  (0.58-0.96) | 0.02 | 1  [Reference] |  |  | - | - | 1  [Reference] |  |  | 1.14  (0.84-1.47) | 0.43 | 0.91  (0.70-1.18) | 0.49 | 1  [Reference] |  |
| **Fear symptoms** | | | | | | | | | | | | | | | | | | | | | | | | | | |
| Total | 0.84  (0.69-1.03) | 0.11 | 1  [Reference] |  |  | 0.87  (0.72-1.05) | 0.16 | 1  [Reference] |  |  | 1.01  (0.82-1.25) | 0.87 | 1  [Reference] |  |  | 1.95  (1.58-2.41) | 0.00 | 1  [Reference] |  |  | 1.01  (0.78-1.29) | 0.92 | 1.32  (1.04-1.67) | 0.03 | 1  [Reference] |  |
| Vaccinated  population | 1.01  (0.72-1.43) | 0.92 | 1  [Reference] |  |  | 0.82  (0.59-1.13) | 0.23 | 1  [Reference] |  |  | 1.12  (0.78-1.59) | 0.53 | 1  [Reference] |  |  | - | - | 1  [Reference] |  |  | 0.82  (0.50-1.33) | 0.42 | 1.54  (0.96-2.47) | 0.06 | 1  [Reference] |  |
| Unvaccinated population | 0.69  (0.41-0.91) | 0.04 | 1  [Reference] |  |  | 0.93  (0.73-1.19) | 0.60 | 1  [Reference] |  |  | 0.96  (0.74-1.26) | 0.80 | 1  [Reference] |  |  | - | - | 1  [Reference] |  |  | 1.12  (0.83-1.51) | 0.45 | 1.21  (0.92-1.60) | 0.16 | 1  [Reference] |  |

Abbreviation: AOR, Adjusted odds ratio; CI, confidence interval.
