# Supplementary material for: Psychological effects and associated factors among vaccinated and unvaccinated general population against COVID-19 infection in Bangladesh
Source: Front Psychiatry. 2022 Aug 12;13:916160. doi: 10.3389/fpsyt.2022.916160 (PMC9412971; doi:10.3389/fpsyt.2022.916160)
Supplement: Supplementary file 3 [file Data_Sheet_1.docx]

**Supplement Figure S1**. Daily COVID-19 related cases in Bangladesh from March 8, 2020, to June 19, 2022 (Data source: Directorate General of Health Services).

**Supplement Figure S2**. Daily COVID-19 related deaths in Bangladesh from March 8, 2020, to June 19, 2022 (Data source: Directorate General of Health Services).

**Supplement Figure S3.** Daily COVID-19 vaccination in Bangladesh from January 27, 2021, to June 19, 2022 (Data source: Directorate General of Health Services).
